# Experimental and Computational Assessment of Adsorbates in Ultraclean 2D MoS_2(1–*x*)_Se_2*x*_ Nanosheets Treated by Ethanol for Enhanced Photodetector Applications

**DOI:** 10.1021/acsanm.5c03601

**Published:** 2025-11-17

**Authors:** Dipak Maity, Ravi K. Biroju, Viliam Vretenár, Mihir Ranjan Sahoo, L’ubomír Vančo, Matej Mičušík, Tharangattu N. Narayanan, Kalpataru Pradhan

**Affiliations:** † Centre for Nanoelectronics & VLSI Design, 382966 Vellore Institute of Technology, Chennai, 600048, India; ‡ 385263 Department of Physics, School of Advanced Sciences, Vellore Institute of Technology, Chennai 600048, Tamil Nadu, India; § Centre for Nanodiagnostics of Materials, Faculty of Materials Science and Technology, 61791 Slovak University of Technology, Vazovova 5, Bratislava, 81243, Slovakia; ∥ Institute of Theoretical and Computational Physics, Graz University of Technology, Graz, Austria, 8010; ⊥ Polymer Institute, Slovak Academy of Sciences, Dúbravská cesta 9, 84541, Bratislava, Slovakia; # Theory Division, 30176 Saha Institute of Nuclear Physics, HBNI, Kolkata, 700064, India; ▼ Surface Science and Interface Engineering Group, Tata Institute of Fundamental Research Hyderabad, Sy. No. 36/P, , Serilingampally Mandal, Gopanpally Village, Hyderabad, 500 107, India

**Keywords:** Semiconducting Transition Metal Dichalcogenides, Wet transfer method, Post transfer cleaning method, Ethanol treatment, Photoresponsivity, Density functional theory

## Abstract

Two-dimensional semiconductor-transition-metal dichalcogenide (2D-STMD) based semiconductors have emerged as promising materials for future spintronic and optoelectronic applications, including photodetectors and transistors. Transferring high-quality chemical vapor deposition (CVD)-grown monolayer 2D-STMDs and their alloys to the target substrate is very challenging for fabricating efficient devices. Unfortunately, current post-transfer methods struggle to completely remove unwanted contamination residues during wet-transfer processes, which adversely affects material quality and intrinsic properties. In this work, the effect of ethanol cleaning on the qualitative and quantitative assessment of molecular adsorbates is demonstrated, based on atomic-resolution high-angle annular dark field scanning transmission electron microscopy (HAADF-STEM) image analysis supported by X-ray photoelectron spectroscopy (XPS), Auger electron spectroscopy (AES), and density functional theory (DFT) calculations, which showcases ultraclean material structures. We estimate the unidentified molecular adsorbates in the proximity of molybdenum (Mo) and chalcogen (S, Se) atomic sites, which are tentatively assigned as ‘C_2_H_5_OH (EtOH)’, ‘H_2_O’, and ‘O_2_’ related adsorbates. The attribution is based on HAADF-STEM Gaussian line shape fitting of atomic intensity columns and corresponding computed adsorption energy values after ethanol treatment of the MoS_2(1–*x*)_Se_2*x*_ (MSSE) alloy. In line with experimental observations of persistent OH-containing residues on the surface, DFT simulations show that EtOH has better adsorption on both pristine and sulfur-vacancy MSSE monolayers than H_2_O and O_2_. Photodetector device measurements revealed a remarkable ∼90% enhancement in photocurrent values for ultraclean samples, significantly boosting the material’s photoresponsivity. DFT calculations on the adsorption energy and density of electronic states were also conducted to validate our experimental findings.

## Graphical abstract

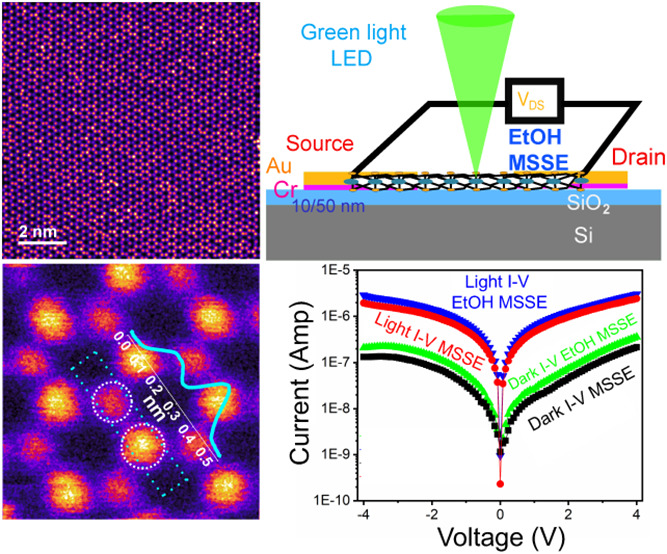

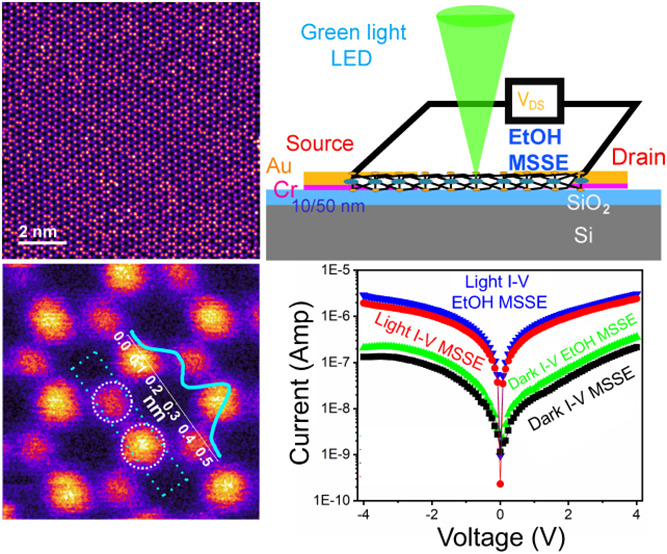

## 1 Introduction

Atomic layer 2D semiconducting transition metal dichalcogenides (2D-STMDs), such as MoS_2_, WS_2_, and MoSe_2_, have garnered significant attention due to their unique electronic and optical properties.^1–4^ Their strong spin–orbit coupling, direct bandgap, and broken inversion symmetry make them suitable candidates for optoelectronics, spintronics, and valleytronic applications.^5–9^ Flexible optoelectronic devices can be achieved by transferring monolayer flakes onto transparent and flexible substrates.^10^ Chemical Vapor Deposition (CVD) is a versatile technique that provides excellent optical quality materials, scalability, and reproducibility for the large-area wafer-scale fabrication of 2D-STMDs.^11–13^ However, the use of several transition metal and chalcogen source materials during the CVD growth of ternary and quaternary alloys makes it more challenging to obtain high-quality samples.^14–16^ On the as-grown samples, bulk contaminants, including salt residues, remain on the surface of the desired 2D-STMD atomic layers.^11^ Therefore, a qualitative and quantitative understanding of such engineered 2D STMD structures is very challenging.^12^ For example, in the case of the 2D ternary MoS_2(1‑x)_Se_2*x*_ (MSSE) alloy, the growth process involves transition metal (TM) and chalcogen sources, such as molybdenum, sulfur, and selenium powders.^16^ After the growth of MSSE, some of the bulk sources may be present on the as-grown MSSE samples. In addition, understanding chemi- and physisorbed species (adsorbates), which may be attached by weak or strong chemical bonds inside the 2D STMD atomic lattice at the TM or chalcogen sites, is crucial in the current 2D STMD research community.

This type of study plays a pivotal role in understanding transport and charge transfer phenomena from various perspectives for electronic, optoelectronic, and catalytic device applications. Here, the CVD growth parameters such as growth temperature, partial pressures, and amounts of the chalcogen mixture (S+Se) and TM (Mo) will vary compared to the growth of pristine structures of MoS_2_ and MoSe_2_ because there is a high probability that source material remnants will also reside on the surface of the as-grown 2D-engineered alloy samples. One can implement pretransfer cleaning techniques, such as plasma treatment, UV irradiation, and annealing (in either vacuum or ambient conditions), to produce high-quality 2D alloy samples for the desired application.^17^ At the same time, these processes may introduce additional surface defects and rearrangement of the atomic structure of the as-grown sample, which may alter the electronic and physicochemical properties of such an engineered 2D layered material.^17^ In the current research community of 2D materials, this is an open question, while implementing these exotic materials with interesting properties for a longer duration in electronic and optoelectronic device applications, including catalysis and photocatalysis, with high-quality 2D material at the nanoscale regime. Note that most of the existing cleaning methods were researched on pristine 2D atomic layers, which may not be implementable for engineered 2D materials such as alloys and van der Waals (vdW) heterostructures (HSs) in an identical manner.^17,18^ There are no reports on the wet transfer cleaning of MSSE in the current research on 2D STMD alloys. However, adsorbent-free 2D STMD is an inevitable and challenging step in any current research. So, postcleaning methods, which follow gentle procedures, are very important to implement on these kinds of exotic layered alloys and HSs of the 2D STMDs.

In past research in this perspective, the direct growth onto flexible substrates, such as polyethene terephthalate (PET) and polyethene naphthalate (PEN), is hindered by high temperatures (>700 °C), necessitating the transfer of CVD-grown samples.^19^ This transfer process is crucial for preparing scanning transmission electron microscopy (STEM) imaging samples and fabricating devices such as vertical 2D heterostructures, CMOS devices, and photodetectors.^20,21^ Among all 2D material transfer methods, the most used techniques are poly methyl methacrylate (PMMA) - based wet transfer, poly dimethylsiloxane (PDMS)-based dry transfer, and metal-assisted transfer.^22^ While metal-assisted transfer offers high adhesion, it faces challenges like mechanical strain.^23^ PDMS-based transfer avoids etchants but leaves polymer residues. PMMA is widely used due to its robustness and flexibility in wet-transfer methods, preserving the structural integrity of 2D atomic layers.^24,25^ However, PMMA residues and postgrowth contaminants persist, negatively impacting the intrinsic properties of STMDs. Residue removal is paramount, as evidenced in graphene research, where PMMA affects mobility, thermal conductivity, and the threshold voltage of the field-effect transistor (FET).^26,27^ Removing PMMA residual contaminants and CVD-grown excess precursor contamination from the postprocessed 2D materials is essential, given its extensive use in various processes, including lithography.^26^ Thus, the extensive use of PMMA in several processes demands an effective cleaning procedure of additional PMMA and other residues to obtain accurate device performance. For example, Ziyuan Lin et al. have recently identified PMMA residues on wet-transferred monolayers of MoS_2_.^23^ Addressing this issue, Liang et al. introduced a localized cleaning method for postlithographic PMMA residues on multilayers of MoS_2_ and WSe_2_ using atomic force microscopy (AFM).^28^ By employing the AFM tip in contact mode, they successfully removed PMMA residues, resulting in significant improvements in FET properties, including enhanced channel conductivity and carrier mobility.^28^ However, this mechanical method is impractical for large-scale applications and continuous use. High-temperature annealing, although effective in some instances, presents challenges in 2D STMDs due to the formation of defects and film oxidation.^29^ Plasma cleaning, efficient for PMMA removal, induces p-type doping in 1L-MoS_2_.^30^ Recently, Bhuyan et al. introduced a novel 96-h ethanol treatment to remove PMMA from monolayer MoS_2_ samples.^17^

Here, we have conducted a systematic study to clean the PMMA and growth residues of atomically transferred layers of MoS_2_ (MS) and MSSE alloy, which are grown on Si/SiO_2_ substrates using the CVD technique. To eliminate excess PMMA residues and postgrowth remnants from the samples, we employed a 100-h hot (30 °C) ethanol treatment and compared the outcomes with the conventional acetone-based PMMA cleaning method.^31^ Before this experiment, we used several techniques, including a 12 h hot acetone treatment and vacuum annealing, to clean PMMA residues. Here, this method is extended to the large-scale cleaning of MSSE alloy atomic layers. The ternary MSSE alloy system provides a more versatile and tunable platform compared to binary MoS_2_ or MoSe_2_. The optical quality of the ethanol-treated ultraclean MSSE is assessed through Raman, optical absorption, and photoluminescence (PL) spectroscopic techniques. The composition of the as-grown samples is assessed via X-ray photoelectron spectroscopy (XPS). High-angle annular dark-field scanning transmission electron microscopy (HAADF-STEM) is employed to quantify the local atomic site-by-site alloy composition in the samples after removing background intensity from PMMA residues/postgrowth adsorbates in the acquired HAADF-STEM image. We proposed the origin of these unidentified molecular adsorbates from C_2_H_5_OH (EtOH), H_2_O, and ‘O_2_’ based on HAADF-STEM and DFT-based calculations. This is further corroborated by XPS and AES spectra analyzed from the free-standing, ultraclean MSSE alloys. HAADF-STEM statistical analysis of alloy atomic composition sites and elimination of ultrathin PMMA residual layer and contamination species adhered to the free-standing MSSE alloy were extensively used to investigate the effect of various treatments. In addition, photodetector devices were fabricated, and their persistent photoconductivity/photoresponse measurements were carried out to evaluate the effect of ethanol treatment on the optoelectronic properties of both pre- and postethanol-treated MSSE alloy. The figures of merit in a photodetector, such as the photoresponsivity and detectivity, are found to be enhanced significantly after ethanol cleaning.

## 2 Experiment

### 2.1 Synthesis of MoS_2_ (MS) and MoS_2(1‑x)_Se_2*x*_ (MSSE)

Monolayer MS and MSSE are synthesized using the powder-assisted chemical vapor deposition (CVD) method, as detailed in our previous studies.^16,30^ In this approach, MoO_3_ (3 mg) served as the precursor material and was placed in an alumina boat. A Si/SiO_2_ substrate with a thickness of 300 nm is positioned on top of the MoO_3_ boat. Sulfur and selenium powders are employed as sources for S and Se. For MoS_2_ growth, the growth temperature on the sulfur side is maintained at 210 °C, while the temperature on the precursor side is set to 710 °C. In the case of MSSE growth, the growth temperatures on the ‘S’ and ″Se″ sides are set to 400 °C, and the precursor side temperature is adjusted to 780 °C. The ramp time and growth time for both processes are optimized at 30 and 15 min, respectively. Nitrogen gas is used as a carrier gas for both growths, with a constant gas flow rate of ∼ 187 sccm.

### 2.2 Transferring of the MS and MSSE Films

For the transfer of the as-grown films situated on the Si/SiO_2_ substrate, we initially coated the substrate with PMMA through a spin-coating process. Subsequently, the coated substrate underwent a heat treatment at 90 °C for 10 min. Following this step, the prepared substrate is immersed in a 2 M KOH solution for 8 h. After the KOH treatment, the film/PMMA layer floating on the solution is carefully washed with water and then transferred onto the desired substrate. To finalize the transfer process, the material is further purified by rinsing it with acetone and water to eliminate the top PMMA layer.

### 2.3 Ethanol Treatment of the MS and MSSE Films

To carry out ethanol cleaning, all the samples and devices were submerged in ethanol at a temperature of 30 °C and left in this solution for 100 h. Subsequently, we performed a cleaning procedure using isopropyl alcohol (IPA) and deionized (DI) water, followed by drying.

### 2.4 Photoconductivity and Photoresponse Measurements

We fabricated devices on Si/SiO_2_ substrates with a thickness of 300 nm by using laser lithography techniques. In this procedure, we applied a positive photoresist, specifically S1813, for coating. Following lithography, a TMAH (13%) developer is used to selectively remove the photoresist from the patterned areas. A thermal evaporator setup is employed for the deposition of Cr and Au (10/50 nm). Subsequently, a lift-off process is executed using acetone, followed by gentle sonication for a duration of 10 s. The thin films are then transferred onto these prefabricated devices through a wet transfer method. All transport measurements are conducted in a probe station setup utilizing the Keithley 2450 source meter. For photoconductivity measurements, a green light LED with a wavelength of 532 nm and a laser power of 4.5 mW/cm^2^ is utilized as the light source. Transfer and output device measurements are performed under ambient conditions at room temperature.

### 2.5 X-ray Photoelectron Spectroscopy

XPS was used for the investigation of elemental chemical states in as-grown MS and MSSE 2D layers. For spectra acquisition, a Thermo Scientific Nexsa G2 analysis system (Thermo Fischer Scientific, UK) with a microfocused monochromatic Al Kα X-ray source (1486.7 eV) was used in a constant analyzer energy regime. Survey spectra were acquired with 1 eV step and 200 eV pass energy, and high-resolution elemental spectra were collected with 0.1 eV step and 50 eV pass energy. To achieve charge compensation, an Ar^+^ flood gun was employed. The surface compositions, presented in atomic percentages, were determined by measuring the integrated peak areas fitted with Gaussian–Lorentzian functions, after subtraction of the Shirley background, and by applying the corresponding sensitivity factors. In the case of Mo, the identified Mo^6+^ species were omitted from MS and MSSE quantification. The spectra were aligned to the C 1s 284.8 eV line.

### 2.6 High Angle Annular Dark Field Scanning Transmission Electron Microscopy (HAADF-STEM)

HAADF-STEM measurements of MS and MSSE before and after ethanol treatment were carried out using an aberration-corrected scanning transmission electron microscope (Jeol JEM-ARM200cF) operated at a low acceleration voltage of 80 kV nondestructively.^32,33^ The convergence semiangle of the incident beam probe was set to 22 mrad. The probe current and size were kept below 16 pA and 80 pm, respectively. The beam damage due to the continuous irradiation of an electron beam is negligible in terms of the formation of ‘S’ vacancies for a few minutes of exposure. The inner and outer semiangles were set at 45 and 180 mrad for collecting STEM images using a HAADF detector, and the images were processed with 64-bit Digital Micrograph GMS 3.44 (Gatan) software. The quantification of HAADF images was performed by open-source Stat STEM software,^34^ as we reported previously.^16^

### 2.7 Auger Electron Spectroscopy (AES)

AES was carried out on a JEOL JAMP-9510F Auger microprobe using 10 keV primary electron energy and 10 nA probe current in a constant retard ratio regime with an energy resolution of 0.6%. The device was equipped with a hemispherical analyzer, and both incident and emission angles were 30. The acquired Auger spectra were evaluated in differentiated mode, followed by 7-point Savitzky-Golay smoothing.

### 2.8 Computational Methodology

The structure optimizations and electronic structure properties of various adsorbates (H_2_O, O_2_, and EtOH) on pristine MoS_2_, MoSe_2_, MSSE and corresponding sulfur vacancy monolayers were calculated within the framework of first-principles density functional theory (DFT) using the Vienna ab initio Simulation Package (VASP) code.^35,36^ The exchange-correlation functional was approximated by Perdew–Burke–Ernzerhof (PBE),^37^ a version of generalized gradient approximation (GGA) within the projector augmented wave (PAW) method. The kinetic energy cutoff of 500 eV was considered for the expansion of plane wave basis sets. Using the Gamma-centered method, the Brillouin zone was sampled with 3 × 3 × 1 and 7 × 7 × 1 k-point grids for self-consistent and density of states calculations (DOS), respectively. A full structure optimization was performed until the Hellmann–Feynman force became less than 0.01 eV/Å for each atom, with an energy convergence threshold of 1 × 10^–5^ eV between successive ionic steps. In this work, we considered a 4 × 4 × 1 supercell (with size 12.724 Å × 12.724 Å × 30 Å) of pristine MSSE and SV_MSSE monolayers to study the adsorption of adsorbate molecules. This size can provide enough space to prevent interactions between the images of adsorbate molecules, which are created due to the periodicity in any direction.

## 3 Results and Discussion

Monolayers of MoS_2_ (MS) and MSSE were synthesized using the CVD method, as detailed in the experimental methods, and a schematic of a CVD growth process is shown in Supporting Information, Figure S1.^16,30^ The ‘Se’ alloying in the MoS_2_ system did not affect the morphology of the crystal, but it did affect the optical bandgap and electronic properties, as reported previously.^16^ An optimal MoS_2(1‑x)_Se_2*x*_ composition (x = 0.34), containing approximately 22% Se, which was reported in our recent work, is synthesized for further investigation.^16^ The as-grown MSSE films are transferred onto Si/SiO_2_ substrates and TEM quantifoil grids, simultaneously using the standard PMMA-based wet transfer method, termed ″before ethanol treatment″ samples. Subsequently, these samples are subjected to 100 h ethanol treatment, referred to as ″after ethanol treatment″ samples as illustrated in the schematic Scheme 1. Figure 1(a) depicts the field-emission scanning electron microscopy (FE-SEM) image of the CVD-grown monolayer crystal of MSSE with a lateral width of around ∼ 50 μm. Ethanol eliminates PMMA and residues through hydrogen bonding with the carbonyl groups of PMMA chains, promoting polymer disentanglement and dissolution without significant swelling. During transfer, ethanol penetrates the PMMA layer, weakening its structure and forming a soluble phase that can be rinsed off, leaving the underlying surface clean.^31^ The mixed chalcogen composition in MSSE introduces higher defect densities, alloy disorder, and nonuniform PMMA interactions during transfer, making it more responsive to cleaning and defect passivation treatments. As a result, the influence of ethanol becomes more pronounced and easier to discern than that in binary systems, where the effect may be subtler. Furthermore, MSSE alloys offer the unique advantage of bandgap tunability through controlled S/Se ratios, making them promising candidates for broadband photodetector applications. Note that the samples and corresponding figures were labeled as ‘MSSE’ and ‘EtOH-MSSE’, respectively, in all the results and discussion sections. The complete experimental details are found in the experimental section. Raman spectroscopy, a versatile tool for 2D-STMD characterization, is employed to assess the structural integrity of MSSE samples. Figure 1(b) displays the Raman spectra of MSSE films before and after ethanol treatment, taken at the same location for a fair comparison. The as-transferred MSSE exhibits A_1g_ (out-of-plane) and E_2g_ (in-plane) vibrational modes at 403.2 cm^–1^ and 377.4 cm^–1^, along with a vibrational peak at 271 cm^–1^ due to partially selenized MoS_2_, as reported in earlier studies.^16,30^ After ethanol treatment, the A_1g_ and E_2g_ modes were red-shifted to 402.7 cm^–1^ and 377.2 cm^–1^, respectively. This redshift may be attributed to the relaxation of compressive strain resulting from the removal of excess PMMA residues.^17^

**Scheme 1:**
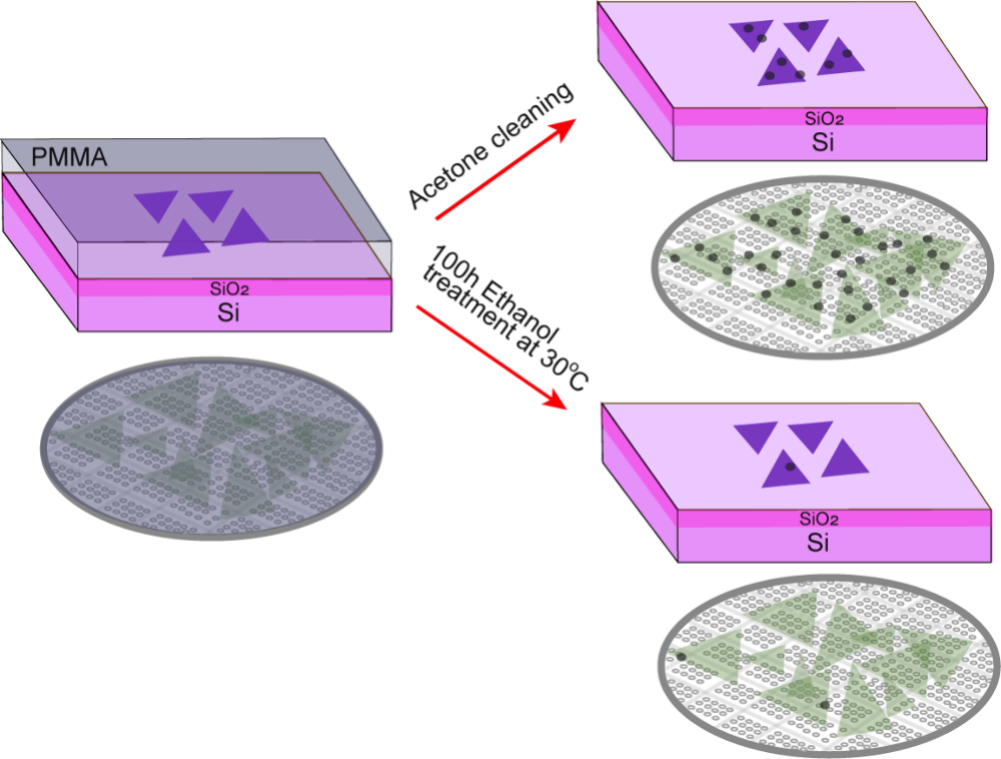
Illustration of Ethanol Treatment Chemistry of MoS_2(1–*x*)_Se_2*x*_ Substrate and TEM Quantifoil Grid with Conventional Acetone Cleaning to Remove the PMMA

**Figure 1:**
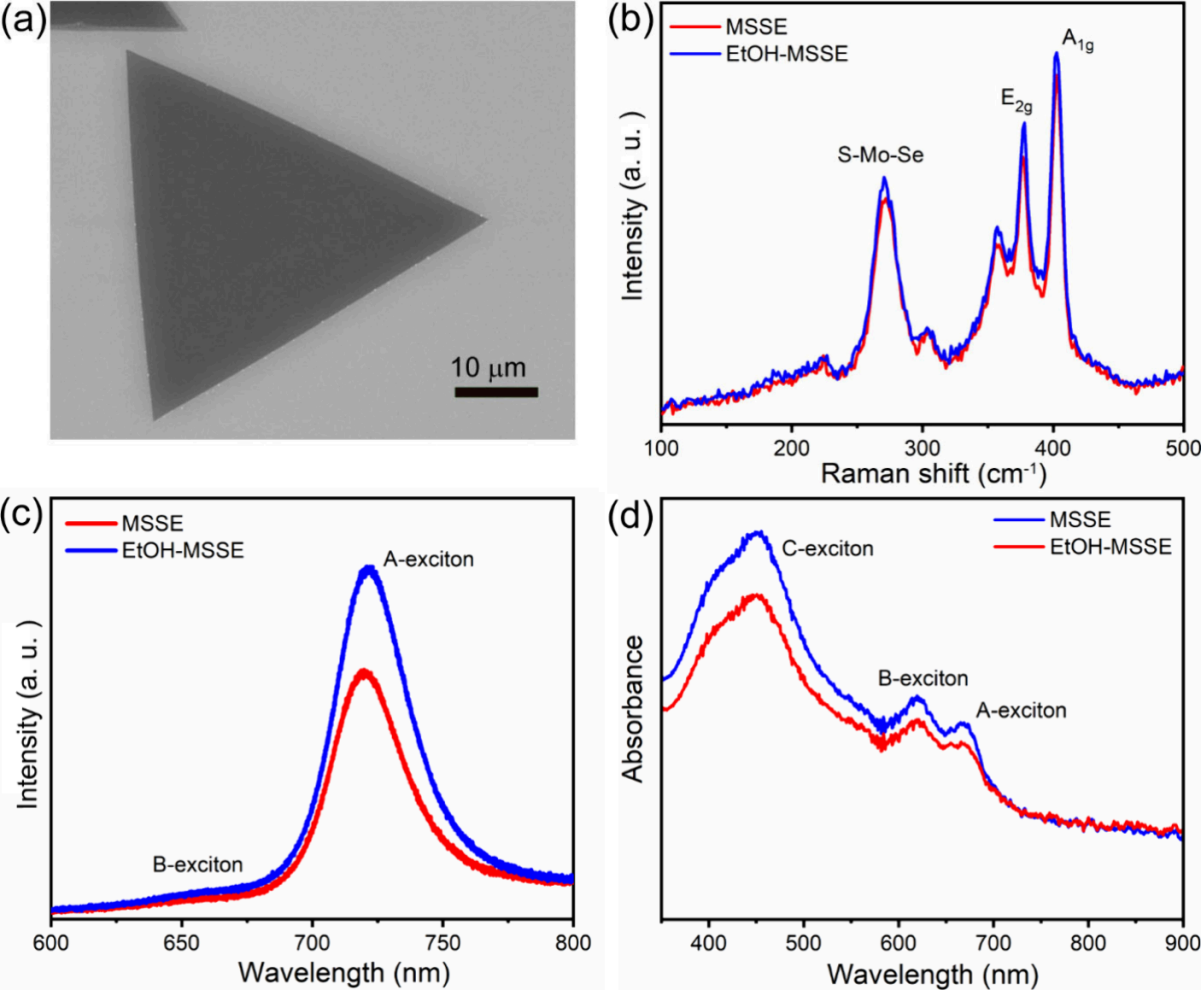
(a) FE-SEM image of an MSSE crystal. (b,d) Comparison of Raman, PL, and optical absorption spectra of MSSE before and after ethanol treatment.

Figure 1(c) presents the photoluminescence (PL) spectra of the before and after ethanol-treated MSSE samples. In the as-transferred film, A-exciton and B-exciton peak positions are found at 720 and 657 nm, respectively. After ethanol treatment, these 2 peak positions were red-shifted to 722 and 659 nm. Alongside the redshift, the PL intensity of the ethanol-treated sample is increased as compared to the as-transferred sample. This redshift and PL enhancement is attributed to the relaxation of compressive strain due to the absence of PMMA residues and the ultraclean nature of the sample.^38,39^ The observed redshift in exciton peaks and increase in intensity may also be influenced by the removal of additional PMMA, as reported in earlier studies,^17,38,39^ indicating the strong influence of the dielectric environment on exciton peaks in monolayer MoS_2_ PL emission. Prior studies on defect repair and PL enhancement in CVD-grown TMDs have been reported.^40–43^ Consistent with these reports, our work also attributes the PL enhancement to defect passivation at chalcogen vacancies, where the vacancies are effectively passivated by dopants or adsorbates. In addition, ethanol treatment in our study helps dissolve the residual PMMA left after transfer, which would otherwise act as nonradiative recombination centers. Figure 1(d) exhibits the absorbance spectra of the MSSE sample before and after the ethanol treatment. Both spectra display A-exciton, B-exciton, and C-exciton, consistent with earlier reports. The absorbance spectra of the ethanol-treated sample showed a slight red shift compared to the untreated sample. This redshift may be attributed to the removal of excess PMMA residues.^17^

XPS measurements were carried out on as-grown MS and MSSE samples to investigate the structural quality and local environment chemical composition of the alloys before and after ethanol treatment. In Figure 2, the XPS analysis is given for as-grown and ethanol-treated MS and MSSE atomic layers residing on Si/SiO_2_. We obtain stoichiometries MoS_2.29_ and MoS_1.59_Se_0.77_ from quantitative analysis (see Supporting Information Table S1) for MS and MSSE, respectively. S-2p spectrum in MS exhibits two standard peaks associated with 2p_1/2_, 2p_3/2_ at 163.9 and 162.7 eV, respectively, with spin–orbit splitting of 1.2 eV. In the case of MSSE, the S-2p spectrum is overlapped with Se-3p components at 166.8 and 161.5 eV with the usual spin–orbit splitting of ∼5.3 eV. The Mo-3d spectrum is similar in both cases, except for the additional Se-3s feature in MSSE. Both spectra are overlapped with the S-2s component and, except Mo^4+^ species at 233 and 229.9 eV, belonging to Mo–S and Mo–S–Se bonds, also exhibit Mo^6+^ oxide peaks at 236.1 and 230 eV. Comparing relative to Mo^4+^ peaks, MSSE contains more ‘Mo’ in the form of oxide than the MS sample. In the Mo-3d spectra of EtOH-MSSE, there is a decrease in intensity for the Mo^6+^ species, which may be attributed to the optimized growth process. Interestingly, there is no significant change in the position of the XPS spectral features, which are already found in the as-grown samples. Further, to investigate the effect of the ethanol treatment and the possible resulting surface modifications in the layers still located on the substrates, we again employed AES. The method enables the reproducible selection of the same location used in the analysis, both before and after the ethanol treatment. For this purpose, we present one MS and one MSSE grain on a Si/SiO_2_ substrate in Figure S3, which were analyzed at three spots prior to and after the alcohol treatment (see Table S2). The spectra of MS prior to treatment exhibit lower carbon contamination than those of MSSE. After the treatment, the carbon peak was significantly enhanced in MS and MSSE, likely due to the introduction of carbon species by thermally active ethanol. The intensity of the Auger peaks of S, Mo, and O decreases in both types of samples after the addition of a carbon overlayer following treatment, but this decrease is more pronounced in the MSSE sample. The decrease diminishes with kinetic energy due to the increase in the inelastic mean free path of Auger electrons, and it already diminishes at ∼ 1300 eV; thus, the Se signal remains constant. Apart from the carbon overlayer, the spectra do not reveal any substantial chemical, structural, or electronic changes; however, one should bear in mind the method’s limited ability to consider these aspects. However, according to the S-LVV and Mo-MNN Auger intensities before and after the treatment seen in Table S2, we may conclude that MSSE is slightly more susceptible to accepting moieties from ethanol than MS. This suggests from both XPS and AES analyses that no chemical change occurs at the chalcogenide or metal sites after ethanol treatment of samples layered on solid substrates. However, a slight formation of new carbon species is observed, which belong to ethanol moieties adsorbed on the surface after treatment, as confirmed by position-dependent AES spectra after ethanol treatment (see Supporting Information, Figure S3). As suggested from the Se-3d spectrum in MSSE, no ‘Se’ oxides are present (expected at 60–57 eV), but some minor extent of ‘Se’ mixed oxides (56–57 eV) cannot be excluded. Also, metallic ‘Se’ states are expected at ∼56–55 eV positions. Thus, we may conclude that ‘Se’ presented in the spectrum is presumably bounded in the selenide. The spin–orbit splitting is 0.85 eV. Higher ‘Se’ content compared to STEM analyses, which will be discussed in the next section, may be caused by the large diameter of the focused excitation primary beam (400 μm), in which case the beam may also excite bulk particles of the deposit in addition to 2D crystals, in favor of ‘Se’ content.

**Figure 2:**
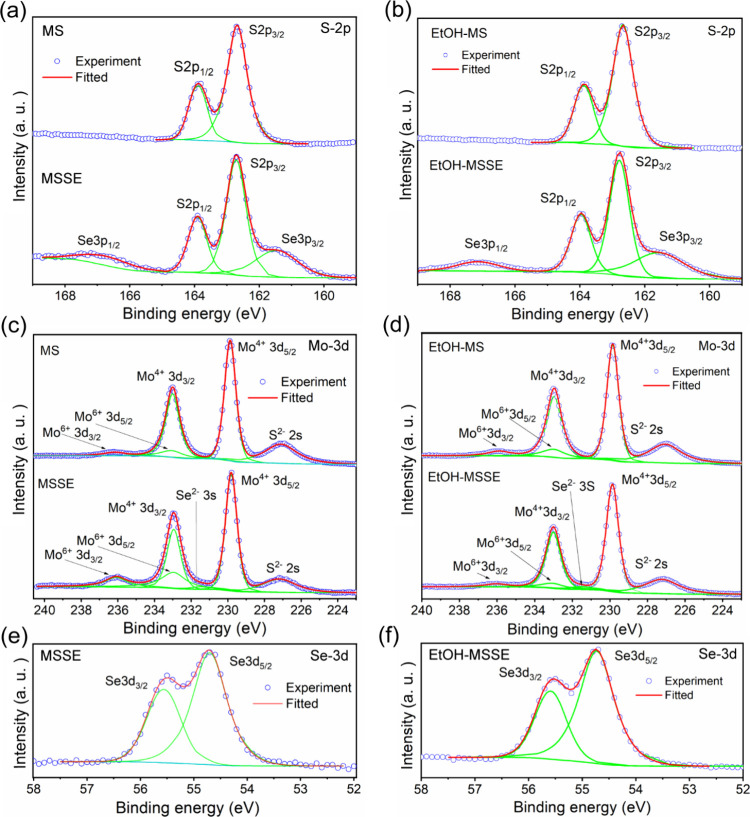
Comparison of XPS spectra in as-grown and ethanol-treated samples of MS and MSSE on Si/SiO_2_ substrates. (a-b) S-2p, (b-c) Mo-3d, and (c-d) Se-3d.

We performed high-resolution HAADF-STEM measurements on both the as-transferred and the ethanol-treated samples to assess the crystalline quality and the effect of ethanol cleaning on the atomic scale. The HAADF-STEM analysis is presented in Figure 3. Figure 3(a,b) clearly shows the presence of PMMA and the postgrowth residues, as indicated by the high background HAADF intensity visible in the transferred samples on the TEM quantifoil grids. A low-magnification HAADF image is shown in the Supporting Information, Figure S4, for further reference. Figure S4­(a-c) clearly depicts the coverage of the polymer/growth residual layer at the intersection of two monolayers, where the vacuum is taken as the reference background and corresponding polymeric and precursor residue intensity (area 2) as well as intensity related to MSSE structure (area 1) are marked in a line profile to visualize the level of contamination adhered to the MSSE monolayer. Figure 3(c,d) shows the HAADF-STEM images of EtOH-MS and EtOH-MSSE, depicted as ultraclean free-standing atomic layers. Additional HAADF-STEM images of ethanol-treated MS and MSSE at different magnifications are shown in the Supporting Information Figure S5. In addition, HAADF-STEM analysis is carried out extensively to demonstrate the highly crystalline nature of the monolayer EtOH-MSSE and the cleanliness of the film at the atomic scale. Ultracleanness of the EtOH-MS sample, as shown in Figure 3(c) in the HAADF-STEM image, is demonstrated by almost zero background without any larger-scale contaminants and the perfect alternation of ‘Mo’ and ‘S_2_’ atomic intensities ratio 1:2, which validates the ethanol cleaning method and is consistent with the previously reported work.^17^ Note that the inset of Figure 3(c) shows the atomic-resolution HAADF-STEM image of a MoS_2_ where lattice sites of ‘Mo’ and ‘S_2_’ can be easily identified due to the Z-contrast of the STEM using high-angle annular dark field HAADF mode (the contrast is approximately proportional to the atomic number Z^1.5–1.8^.^16^ Figure 3(d) represents the atomic-resolution STEM image of an EtOH-MSSE sample without using any background removal filter. In the case of as-transferred MS and MSSE, a thick background intensity is visible as a function of the HAADF intensity of the atomic sites. In other words, hot ethanol treatment is beneficial for the cleanliness of MSSE 2D atomic-layered samples. We did not observe any structural degradation or chemical damage to the 2D materials, as confirmed by HAADF-STEM. HAADF-STEM images were acquired on multiple samples, and the images were acquired at multiple locations in different regions of interest in the same sample. The accuracy of the results may be compromised if we acquired HAADF-STEM images at the same location, due to continuous electron beam exposure that can cause beam damage and potentially generate additional ‘S’ vacancies knocked out of the S atoms, including additional carbon contamination resulting from electron beam irradiation. This ethanol treatment procedure is applicable to any 2D system to remove the PMMA residue during the wet transfer process.

**Figure 3:**
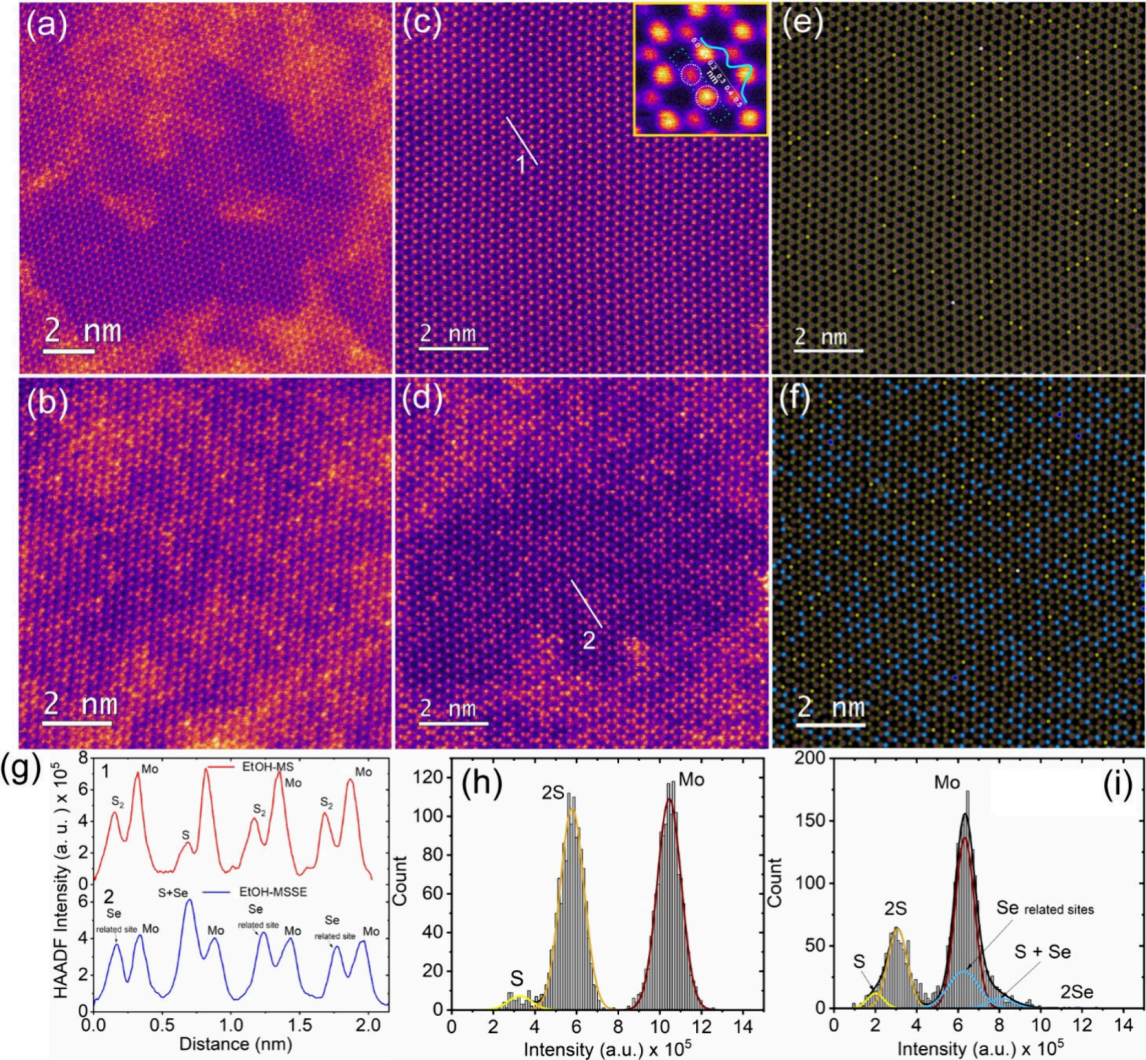
HAADF-STEM image analysis of pristine MS and alloyed MSSE before and after ethanol treatment: (a) MS and (b) MSSE before ethanol treatment and HAADF–STEM images of (c) EtOH-MS and (d) EtOH-MSSE after ethanol treatment. Note that the inset of Figure 3(c) shows an atomically resolved ultraclean MoS_2_ HAADF image and corresponding Gaussian line profile of ‘Mo’ and ‘S_2_’ atomic sites. Corresponding atomic structure models of (e) EtOH-MS and (f) EtOH-MSSE. (g) HAADF intensity line profiles correspond to the marked regions 1 and 2 in Figure 3(c,d), respectively. Gaussian line shape analysis of (h) EtOH-MS, and (i) EtOH-MSSE intensity histograms showing the fitting of atomic compositions corresponding to ‘Mo’, ‘2S’, ‘2Se’, and ‘S+Se’, including unwanted adsorbates with unknown atomic composition ‘Se’.

Figure 3(e,f) shows the fitted atomic structure model of the corresponding HAADF STEM images that are shown in Figure 3(c,d), respectively. Note that the simulated HAADF-STEM images of Figure 3(e,f) are shown in the Supporting Information Figure S6. The structural model of both EtOH-MS and EtOH-MSSE was processed from Gaussian line shape analysis using Stat-STEM software, as detailed in the experimental section. The quantitative analysis of the EtOH-MS structure model (see Supporting Information Table S3) proves the atomic composition of Mo:S is 1:2, which is a significant figure of merit with respect to the local density of atoms and ‘S’ vacancy defects from a large scanning area point of view. Figure 3(c) shows a completely intact hexagonal lattice structure of ‘Mo’ atoms alternating with ‘S’ sites (each having two ‘S’ atoms on top of each other); corresponding atomic sites of ‘Mo’ and ‘S’ are indicated with yellow and red colors, respectively, in the structure model (see Figure 3(e)). Note that the bright yellow and white colored sites represent the single and double sulfur vacancies, respectively. Further, we have extended this statistical analysis on the MSSE alloy sample after the purification process to achieve qualitative results. Figure 3(g) shows line profiles through the two locations extracted from regions 1 and 2 as shown in Figure 3(c,d), respectively. Profile 1 contains three alternations of ‘S_2_’ and ‘Mo’ and one alternation of ‘S’ and ‘Mo’ atoms. Profile 2 depicts the three alternations of ‘unknown adsorbates’ at the chalcogenide atomic site, consecutive “Mo” atoms, and one alternation of ‘S+Se’ site and ‘Mo’ site. Interestingly, in EtOH-MSSE, we observed an unambiguous difference in the atomic site positions of the alloy. Specifically, alongside the S+Se, new atomic site intensities emerged after ethanol treatment, as described in the line profile of EtOH-MSSE. The HAADF intensities are almost equal compared to ‘Mo’ atoms, which were noticed at both the metallic and chalcogen atomic sites (see Figure 3(g)). According to our hypothesis, this might be due to the adsorption of ‘H_2_O’ or ‘O_2_’ or ‘EtOH’ species at the sulfur vacancy chalcogen atomic sites, which may be in the form of ‘Se+OH’, which leads to the variation of HAADF intensities corresponding to the ‘S+Se’ atomic sites.^16^ It was reported that out of ‘O_2_’ or ‘H_2_O’ or ‘EtOH’ molecular adsorbates ‘EtOH’ adsorption is the most likely component to be adsorbed on defective STMD, such as MoS_2_, due to ‘EtOH’ possessing low adsorption energy.^33^ Without quantitative statistical analysis, however, atomic identifications such as these are rather tentative. The appropriate way to quantify the image is to compute a histogram showing the distribution of the atom intensities^16^ for all the atoms in a given area and to use the histogram to determine the probability that the atomic assignments were made correctly. Such an analysis seems not to have been done before on large-area HAADF-STEM images of an MSSE alloy. We attempt to further evaluate the intermittent atomic site intensities at the chalcogenide HAADF atomic sites. We have extensively simulated the ultraclean EtOH-MS and EtOH-MSSE HAADF intensities, which were fitted with a Gaussian function as depicted in Figure 3(e,f), respectively. Corresponding Gaussian line-shape fitting profiles of the atomic composition are shown in Figure 3(h,i).

Interestingly, we have found that there may be the presence of oxides (O_2_) or H_2_O or EtOH based on the Gaussian fitting of HAADF intensities, particularly at the chalcogen atomic sites on the basal plane of MSSE after the 100 h treatment with ethanol in the free-standing atomic layers. There is an ambiguity of whether the atomic structure of EtOH-MSSE alloy is altered with the molecular species of ‘H_2_O’, ‘O_2_’, or ‘EtOH’ related adsorbates due to the treatment with the water-containing ethanol,^44^ leading to the rearrangement of the atoms. In addition, as-grown MSSE samples are also influenced by the residues of precursors used for CVD growth in the present case. We previously discussed in detail the use of HAADF-STEM imaging analysis on EtOH-MSSE samples. This type of study requires immediate attention, as the proposed cleaning methods may yield partially improved results for MSSE alloys compared to the conventional treatment methods reported for pristine 2D STMDs. To support our hypothesis further, we performed AES measurements of ethanol-treated MS and MSSE samples transferred onto the TEM quantifoil grids as shown in Figure 4. Figure 4(a) displays a comparative AES spectrum of EtOH-MS and EtOH-MSSE free-standing 2D atomic layers, and corresponding scanning areas are represented by SEM images in Figure 4(b,c), respectively. Note that the spectra were recorded only from the areas with the holes manufactured in the supporting carbon quantifoil, that is, where the 2D layers were suspended over vacuum, and each spectrum was compared to the 3 consecutive holes as shown in Figure 4(b,c). Hereby, the O-KLL signal adsorbed on supporting carbon foil or any substrate can be excluded, enabling assignment of oxygen species affiliated directly to the 2D atomic layer. Spectra in both types of samples contain transitions S-LVV (150 eV), Mo-MNN (184 and 224 eV), and C-KLL (272 eV). Carbon signals come most probably from organic moieties after ethanol treatment or from CVD growth remnants after PMMA transfer. In addition, the EtOH-MSSE sample also contains Se-LMM (1205, 1311, and 1350 eV) Auger transitions as expected. What is the most significant difference between EtOH-MS and EtOH-MSSE spectra is the intensity of O-KLL (505 eV) transitions. While in the EtOH-MS sample, it is barely legible and close to the detection limit, in the MSSE sample, the O signal is much more intense. Since there is no supporting substrate to take effect in the spectra, the O in MSSE is connected directly with the layers themselves. This corroborates the hypothesis drafted from the STEM analysis that Se sites are most probably modified by some oxygen-containing molecules/radicals.

**Figure 4:**
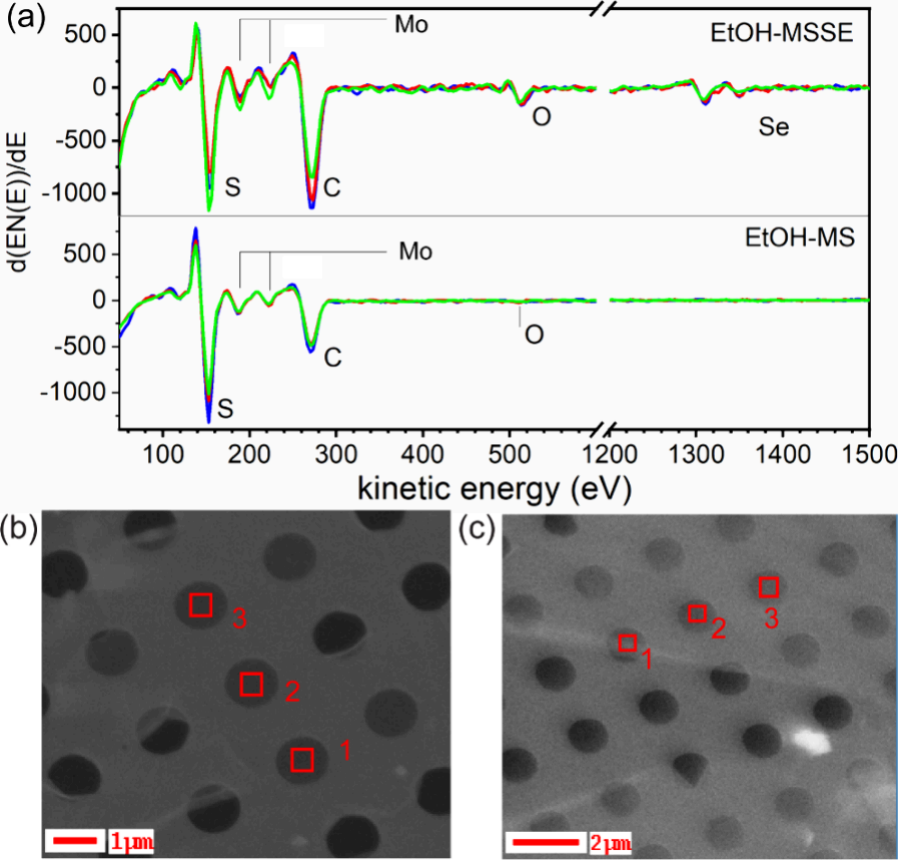
(a) Auger electron spectra from three different monolayers suspended over the holes (1 μm in diameter) patterned in a TEM quantifoil grid, measured in ethanol-treated MS/MSSE samples. (b,c) Corresponding SEM images of the holes where MS and MSSE atomic layers are free-standing on the TEM quantifoil, respectively.

Photoconductivity (PC) and photoresponse measurements were conducted to assess the impact of ethanol cleaning on the MSSE-based photodetector devices. The fabrication and measurement procedure for preparing devices is outlined in the experimental section. A schematic of the experimental setup is shown in Figure 5(a), illustrating photodetection from an ultracleaned EtOH-MSSE alloy. Note that the inset shows the optical microscope image of the ultracleaned EtOH-MSSE alloy, and the zoomed version of the device shows the perfect Cr/Au contacts over the EtOH-MSSE device. To examine the cleaning process’s effects, current–voltage (I–V) characteristics of EtOH-MSSE devices are measured before and after ethanol cleaning under dark conditions and light exposure, utilizing green laser (532 nm) excitation. Figure 5(b) presents a comparative analysis of steady-state dark current and photocurrent as a function of voltage for MSSE samples before and after ethanol treatment. Before PC measurements, all samples are stored under vacuum in the dark overnight to ensure complete relaxation. Photocurrent (I_ph_) values are calculated using the formula I_ph_ = I_light_ – I_dark_.^39^ The I–V curves exhibited linearity and symmetry in the −1 V to +1 V bias regions, confirming ohmic contact between the measured samples and Cr/Au electrodes. Here charge transport takes place from the Cr/Au electrode to MSSE and returns to the Cr/Au electrode. Notably, the photocurrent value increased after the ethanol cleaning process, as observed in Figure 5b. This improvement in photoconductivity is attributed to removing PMMA residues and CVD growth remnants during ethanol cleaning, which may act as scattering centers for charge carriers. Additionally, the time response curves as a function of the bias voltage are obtained under 532 nm excitation at a laser power of 4.5 mW/cm^2^. Figure 5(c,d) shows the comparison of cyclic photoresponse for the devices before and after ethanol treatment at four different external bias voltages: 0.5, 1, 2, and 3 V, respectively. The cyclic response shows the reproducibility of the photocurrent in both cases. Before ethanol treatment, the MSSE device showed a photocurrent value of 0.79 μA at a bias voltage of 3 V. After ethanol treatment, the photocurrent value increased to 1.51 μA at a bias voltage of 3 V, representing a 90% increase in photocurrent in the case of the EtOH-MSSE alloy. The increment in persistent photocurrent is attributed to the removal of PMMA and precursor residues from the MSSE alloy after ethanol treatment. The key parameters, such as photocurrent, responsivity, response time, and detectivity of the MSSE device, and related 2D heterostructures are as discussed in previous studies.^16,45–47^ The photoresponsivity (R) value is estimated as a function of bias voltage before and after the ethanol-treated EtOH-MSSE sample. Table 1 represents the calculation of ‘*R*’ values at each bias voltage in both cases; the corresponding equations used for this calculation are shown below. ‘*R*’ as a function of light intensity is calculated using the equation,

1 $$R=\frac{I_{ph}}{P\times A},\left(Amp/W\right)$$

where ‘*I*_*ph*_’ is the photocurrent, ‘*P*’ is the total incident optical power, and ‘*A*’ is the effective illumination area. Before ethanol treatment, the MSSE device shows a responsivity value of 8.8 × 10^3^ mA/Watt, at the bias voltage of 3 V. After ethanol treatment the value of responsivity is 16.7 × 10^3^ mA/Watt, at the bias voltage of 3 V; i.e., like photocurrent the value of responsivity is increased ∼90% in the case of EtOH-MSSE. The increment in responsivity is attributed to the removal of extra PMMA residues, including CVD growth precontaminants present on the MSSE alloy after ethanol treatment. A summary of the electrical measurements, both before and after ethanol treatment, is presented in Table 1. From both optical and electrical characterization, as discussed above, it can be concluded that there is a significant improvement in film quality following PMMA removal after ethanol treatment. The effect of cleaning for large-area MSSE alloys is clearly reflected in the photoconductive device performances. Therefore, this work will enable us to make a next-generation high-responsive photodetector using an MSSE alloy.

**Figure 5:**
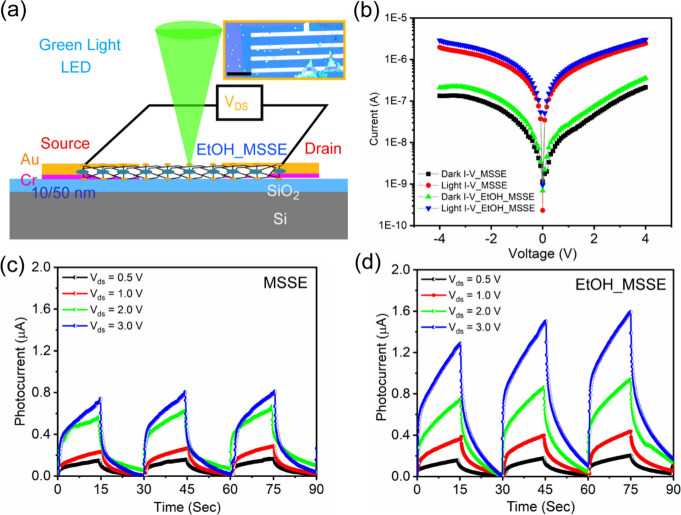
(a) Schematic of the photodetector device using EtOH-MSSE and inset shows an optical microscope (OM) image of the fabricated PD device of EtOH-MSSE. Scale bar in the inset of the OM image is 10 μm. (b) Dark and Light I–V characteristics of the MSSE before and after the ethanol treatment. Photocurrent and Photoresponse of the (c) MSSE and (d) EtOH-MSSE sample.

**Table 1:** Comparison of Device Performance before and after Ethanol Treatment

	Photocurrent (μA)		Responsivity (×10^3^ mA/W)		Detectivity (×10^10^ Jones)		Response time (sec)	
Bias Voltage (V)	before	after	before	after	before	after	before	After
0.5	0.15	0.19	1.6	2.1	12.1	21.2	11.6	11.2
1	0.27	0.41	3.0	4.6	18.7	29.8	11.4	11.1
2	0.60	0.87	6.7	9.7	27.9	35.6	11	10.8
3	0.79	1.51	8.8	16.7	28.1	47.5	12	11.6

Further, we investigated the adsorption of molecules on pristine and S- and Se-terminated monolayers of the MSSE system, as shown schematically in the Supporting Information, Figure S7. For each molecule, there are three possible sites for adsorption, which are called hollow, Se, and Mo sites, depending upon the location of the center of the adsorbate molecules. In addition, we considered both the S-terminated and Se-terminated sides of the S_V_-MSSE monolayer, and we have calculated the adsorption energy (*E*_ads_) as shown in the equation below

2 $$E_{\mathrm{ads}}=E_{\mathrm{surf}+\mathrm{mol}}-E_{\mathrm{surface}}-E_{\mathrm{mol}}$$

where ‘E_surf+mol_’ is the total energy of the system consisting of pristine/defect monolayer and molecule, whereas ‘E_surf_’ and ‘E_mol_’ represent the total energies of the monolayer and isolated molecule, respectively. A more negative ‘*E*_ads_’ value indicates a stronger interaction between the molecule and the surface. The adsorption energies of different species on both pristine and defective (sulfur-vacancy, S_V_) MSSE monolayers, considering both the S- and Se-terminated sides. Figure 6 shows the adsorption energies of expected adsorbate molecules such as C_2_H_5_OH (EtOH), H_2_O, and O_2_ in the case of S-terminated and Se-terminated surfaces of pristine MSSE and S_V_- MSSE monolayers. Figure 6(a,b) represents a comparative estimation of the adsorption energies of EtOH, H_2_O, and O_2_ in pristine MSSE and S_V__MSSE monolayers at the Mo, S, and hollow atomic sites on the surface of the alloy. On the S-terminated side of both monolayers, H_2_O and O_2_ molecules exhibit weaker binding to the surface than ethanol (EtOH). For the pristine MSSE surface, the adsorption energies of H_2_O (and O_2_) at the hollow, Mo, and S sites are −0.16 eV (−0.11 eV), −0.15 eV (−0.13 eV), and −0.11 eV (−0.05 eV). In contrast, EtOH shows stronger binding at all sites. The adsorption energies for EtOH on the pristine MSSE (S_V__MSSE) surface are −0.30 eV (−0.29 eV), −0.30 eV (−0.28 eV), and −0.27 eV (−0.26 eV) for the hollow, Mo, and S sites. A similar trend is observed when the molecules are adsorbed on the Se-terminated side of both monolayers, as shown in Figure 6(b). The corresponding adsorption energies are slightly higher than those on the S- and Se-terminated surfaces. Notably, the EtOH molecule binds more strongly with an absorption energy of −0.35 eV at the Mo site for both the pristine and S_V__MSSE monolayers. Overall, the differences in adsorption energies between the pristine and defective surfaces are minimal, which aligns well with our experimental observations.

**Figure 6:**
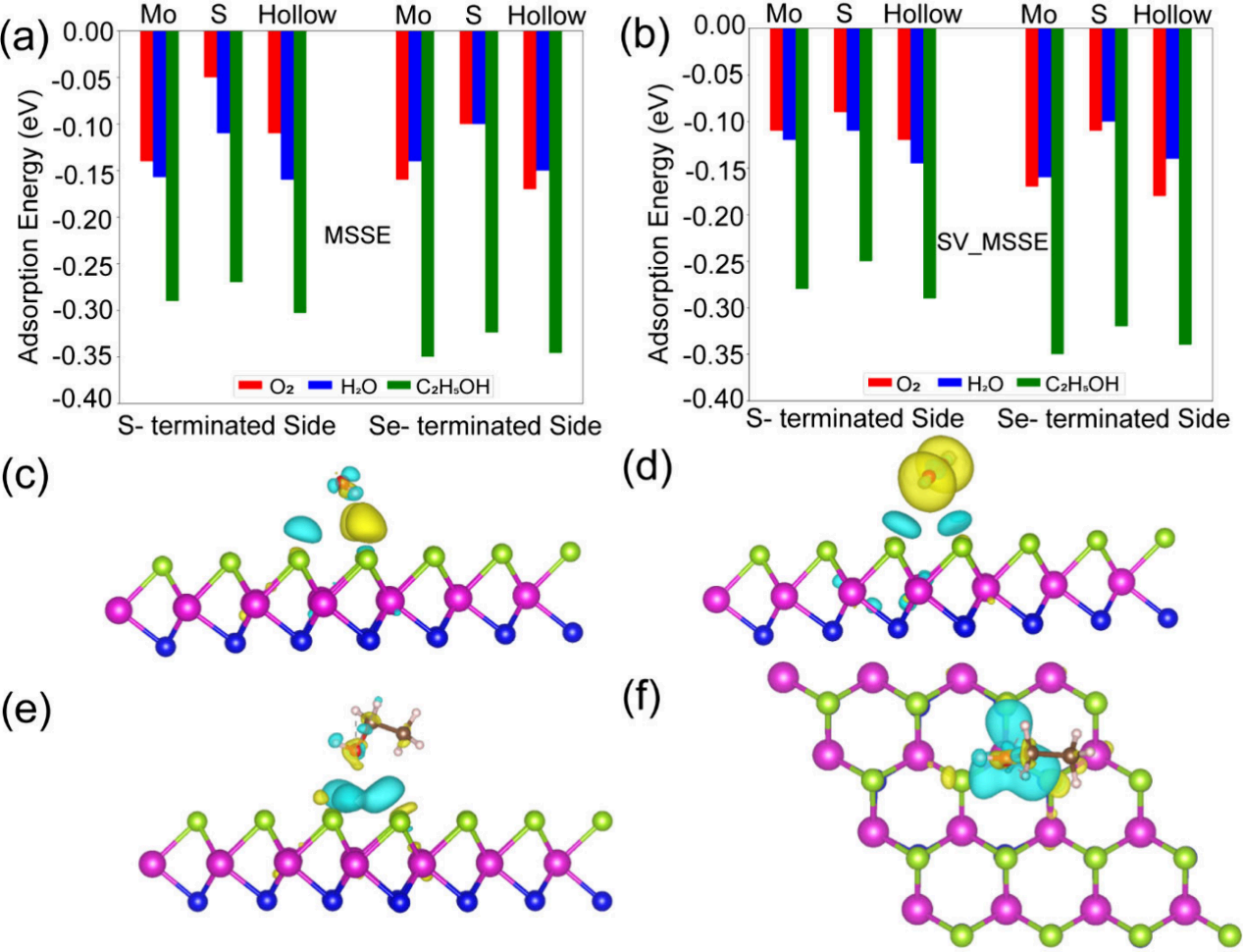
Adsorption energies of C_2_H_5_OH, H_2_O, and O_2_ molecules on (e) S-terminated and (f) Se-terminated surfaces of both pristine MoS_2(1‑x)_Se_2*x*_ (MSSE) and sulfur-vacancy MoS_2(1‑x)_Se_2*x*_ (SV_MSSE) monolayers. Each bar corresponds to the total adsorption energy for a given molecule. (a) Side view of the charge density difference between SV_MSSE and an adsorbed H_2_O molecule. (b) Side view of the charge density difference between SV_MSSE and an adsorbed O_2_ molecule. (c, d) Side and top views of the charge density difference between the EtOH molecule and the SV-MSSE monolayer. Yellow and cyan lobes represented electron accumulation and depletion regions, respectively (isosurface = 0.0005 e/Å^3^). Mo, S, Se, O, C, and H atoms are depicted as magenta, blue, green, red, brown and white spheres, respectively.

In order to explain the difference in interaction strength of different adsorbate molecules with the pristine/defective MSSE monolayers, we have calculated the charge density difference ((Δρ­(r) = ρ_surf+mol_ – ρ_surface_ – ρ_mol_) for visualization of the electron transfer between the surface and the adsorbate. Figure 6(c-e) represents the side view of the charge density difference between Sv_MSSE and an adsorbed molecule such as H_2_O, O_2_, and EtOH. The top view of the charge density difference between EtOH molecule and S_V_-MSSE is also shown in Figure 6(d). When the ‘EtOH’ species is adsorbed onto the surface, a significant charge redistribution is observed compared to the adsorption of ‘H_2_O’ and ‘O_2_’ molecules as depicted in Figure 6(c,d). Specifically, a higher electron accumulation region is observed around the “O” atom of the hydroxyl group (OH), indicating substantial charge transfer and stronger interaction. Conversely, the primary electron depletion site is the Se atom of the surface bonded to the OH species. Additionally, quantitative charge analysis was performed using Bader charge analysis.^48^ Upon ‘H_2_O’ molecule adsorption onto the SV_MSSE monolayer, the O atom gains 1.22 e charge, while the average charge on the ‘H’ atom is 0.6 e. Consequently, a minimal charge transfer of 0.02 e occurs from the monolayer to the ‘H_2_O’ molecule. For ‘O_2_’ adsorption, a slightly higher amount of electron (0.16e) is transferred from the surface to the adsorbate molecule. However, in the case of ‘EtOH’ adsorption, a significant charge redistribution occurs, strengthening the interaction between the adsorbate and the surface. Specifically, one C atom loses 0.37e, the O atom gains 1.13e, and one H atom loses 0.64e, indicating strong charge transfer and bonding. A similar trend is also observed for probing the MSSE monolayer, suggesting a consistent interaction behavior across both surface types.

To gain further insight into the surface interactions, we computed the adsorption energies of EtOH (C_2_H_5_OH), H_2_O, and O_2_ molecules on pristine and chalcogen vacancy (S_V_ and Se_V_) MoS_2_ and MoSe_2_ monolayers as shown in the Supporting Information, Figure (S8–S9), and corresponding optimized atomic models of adsorbates such as EtOH, H_2_O, and O_2_ are shown in the Figure (S8–S9) at three different adsorption sites: the transition metal atom (Mo), the chalcogen atom (S or Se), and the hollow site (center of the hexagonal ring). As illustrated in Figure (S8–S9), MoSe_2_ exhibits slightly stronger adsorptions for all three molecules than MoS_2_, suggesting enhanced molecular interaction with the MoSe_2_ surface. Among the molecules studied, C_2_H_5_OH consistently shows the strongest adsorption across all sites and on both pristine and defective monolayers, with adsorption energies significantly lower (i.e., more negative) than those of O_2_ and H_2_O. This trend indicates a greater affinity of EtOH for these 2D surfaces. Interestingly, for both MoS_2_ and MoSe_2_, the adsorption of C_2_H_5_OH is notably stronger at the Mo and hollow sites compared with the chalcogen (S or Se) sites. This site-dependent preference highlights the role of local coordination environments in governing molecular binding. The stronger interaction at these sites may facilitate the retention of C_2_H_5_OH residues on the surface. This phenomenon aligns well with our experimental observations and reported values of residual ethanol adsorption on the samples following wet chemical processing or exposure.^49^ As illustrated in Figure S10, introducing chalcogen vacancies enhances molecular adsorption in both systems, with Se-vacancy MoSe_2_ showing overall stronger interactions compared to S-vacancy MoS_2_. Among the molecules studied, C_2_H_5_OH exhibits the most favorable (i.e., most negative) adsorption energies across all adsorption sites and for both defective monolayers, indicating its strong affinity toward these surfaces. Notably, the Mo site and the hollow site consistently emerge as the most favorable binding locations for C_2_H_5_OH in both systems. This site-specific trend emphasizes the critical role of local coordination and defect-induced electronic states in governing adsorption behavior. The enhanced binding observed, particularly in defective MoSe_2_, may explain the increased retention of ethanol residues on the surface following wet chemical processes, as was also seen in our experimental studies. Overall, these results underscore the substantial impact of chalcogen vacancies on the molecular adsorption properties of MoS_2_ and MoSe_2_ monolayers with implications for applications in sensing, catalysis, and surface functionalization.

Further, based on the orbital interaction between adsorbate and the SV_MSSE monolayer, we have computed the total and projected density of states (PDOS) of the surface with adsorbates, as shown in Figure 7. Note that the PDOS of the adsorbate-free MSSE is shown in the Supporting Information Figure S11. In the case of H_2_O and EtOH adsorptions, the conduction band region is primarily dominated by the orbitals of the Mo, S, and Se atoms, with no significant contribution from the adsorbates. However, below the Fermi level, clear contributions from the p-states of the adsorbate species are observed. When an ‘H_2_O’ molecule is adsorbed, its ‘O-p’ states lie far below the Fermi level and may hybridize with the ‘Se-p’ states, resulting in a weak interaction. In the case of ‘O_2_’ adsorption, although the ‘O-p’ states appear near the Fermi level, they do not significantly overlap with any states of the SV-MSSE monolayer. Upon EtOH adsorption, the hydroxy group contributes noticeably to the total density of states around 2 eV below the Fermi level by hybridizing with the P-states of the S and Se atoms in the monolayer. The orbital delocalization of the ‘OH’ species enhances its interaction strength with the surface, leading to stronger adsorption compared to other adsorbates.^50,51^ This observation aligns well with experimental findings, where ‘OH’ residues predominantly remain on the surface.

**Figure 7:**
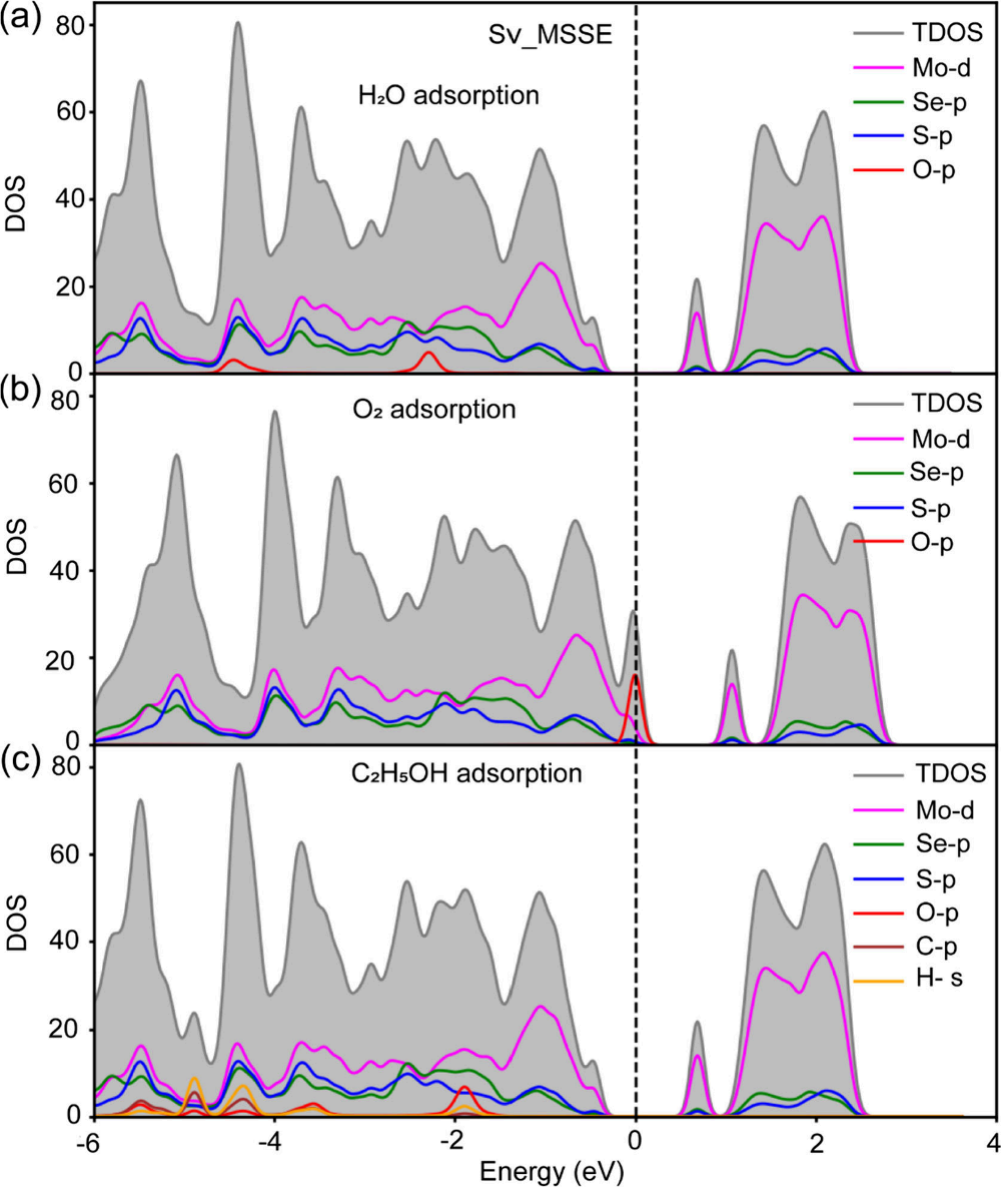
Total and projected density of states of various adsorbates (H_2_O: top, O_2_: middle, and EtOH: bottom panels) adsorbed on a sulfur vacancy MSSE (SV_MSSE) monolayer. The Fermi level is set at 0 eV.

In addition, we further investigated the interaction by positioning the adsorbate molecules directly at the S-vacancy site on the SV-MoSSe surface. For ethanol (EtOH), the molecule becomes significantly distorted as the OH group binds to the S-vacancy site and is separated from the rest. In the case of the H_2_O molecule, the adsorption energy is relatively low at – 0.25 eV, indicating weak interaction. However, for O_2_, when one of its O atoms is placed at the S-vacancy site (as shown in Figure 8(a)), the adsorption energy increases significantly to – 3.0 eV (see Figure 8(b)), suggesting a much stronger interaction. The density of states (DOS) analysis reveals pronounced hybridization between the O-2p orbitals of O_2_ and the Mo-d and S-p orbitals of the substrate just below the Fermi level (Figure 8(b)), further confirming the strong bonding. This result correlates well with experimental observations, where only a small number of the O_2_ residues are detected on the SV-MoSSe surface, likely due to their strong and possibly irreversible adsorption at vacancy sites.

**Figure 8:**
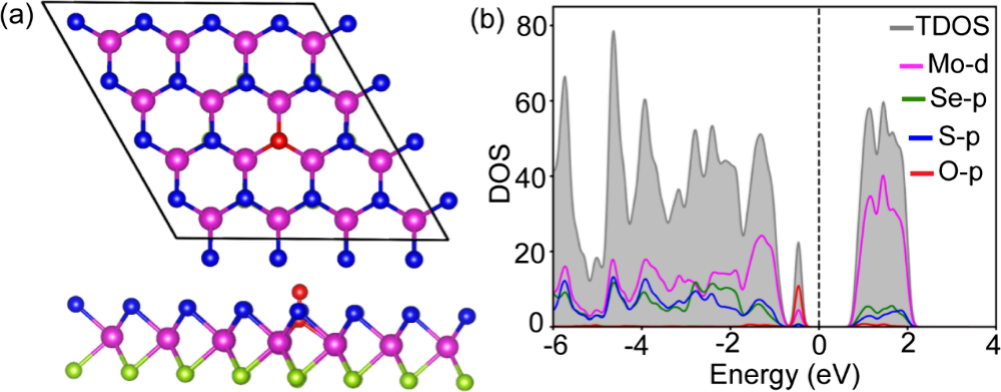
(a) Top and side views of the O_2_ molecule adsorbed at the S-vacancy of the SV_MSSE monolayer. (b) Total and projected DOS of the O_2_ molecule adsorbed at the S-vacancy of the SV_MSSE monolayer (Fermi level is set at 0 eV).

## 4 Conclusion

In the present study, monolayer MSSE alloys are grown using a CVD method. A simple and highly effective ethanol treatment (100 h at 30 °C) is employed to clean PMMA and CVD growth residues during the transfer process, and the results are compared before and after ethanol treatment. Detailed Raman, PL, and optical absorption measurements are performed to ensure the optical quality of the ethanol-treated MSSE samples. The effect of PMMA cleaning is further verified by using extensive HAADF-STEM, XPS and AES-based analysis. Our DFT analysis supports the experimental findings of improved surface retention of OH-functional species by demonstrating that EtOH binds more strongly due to considerable charge transfer and orbital hybridization with MSSE monolayers. Moreover, photodetector-based device measurements are performed to see the effect of ethanol cleaning on the MSSE samples. An enhancement of ∼ 90% in the values of persistent photocurrent and photoresponse is observed after ethanol treatment, revealing the effectiveness of the ethanol-cleaning process. In summary, this work will enable the making of a next-generation high-responsive photodetector using a high-quality MSSE including other 2D alloys.

## Supplementary Material



## Notes

Schematic of a CVD growth of MoS_2_ and Mos_2(1‑x)_Se_2*x*_ nanosheets, Survey XPS spectra of as grown MoS_2_ and Mos_2(1‑x)_Se_2*x*_; HAADF-STEM image of Mos_2(1‑x)_Se_2*x*_ before and after Ethanol treatment; HAADF-STEM images of Ethanol cleaned MoS2 and Mos_2(1‑x)_Se_2*x*_ at different magnifications, such as 10, 15, and 20 Mx; A quantitative HAADF-STEM image analysis using Stat-STEM software; Schematic atomic models of pristine and sulfer-vacancy (SV_MSSE) and corresponding adsorption sites on both S- and Se- terminated surfaces; Comparison of adsorption energies of various molecules on pristine and chalcogenide vacancy defect (Sv and Se_V_) MoS_2_ and MoSe_2_ monolayers; Optimized structures of various molecules adsorbed on pristine and chalcogen vancancy defect MoS_2_ and MoSe_2_; Total and projected density of states in adsorbate free SV_MSSE (PDF)
