# Supplementary material for: Experimental and Computational Assessment of Adsorbates in Ultraclean 2D MoS2(1–x)Se2x Nanosheets Treated by Ethanol for Enhanced Photodetector Applications
Source: ACS Appl Nano Mater. 2025 Nov 17;8(47):22573–85. doi: 10.1021/acsanm.5c03601 (PMC12670367; doi:10.1021/acsanm.5c03601)
Supplement: Supplementary file 1 [file an5c03601_si_001.pdf]

Experimental and Computational Assessment of Adsorbates in Ultra-Clean 2D $MoS_{2(1-x)}Se_{2x}$ Nanosheets Treated by Ethanol for Enhanced Photodetector Applications

Dipak Maity[1$], Ravi K. Biroju[2, 3$*], Viliam Vretenár[3], Mihir Ranjan Sahoo[4], Ľubomír Vančo[3], Matej Mičušík[5], Tharangattu N. Narayanan[1], and Kalpataru Pradhan[6]

[1]Surface Science and Interface Engineering Group, Tata Institute of Fundamental Research Hyderabad, Sy. No. 36/P, Serilingampally Mandal, Gopanpally Village, Hyderabad, 500 107, India.
[2]Centre for Nanoelectronics & VLSI design and Department of Physics - School of Advanced Sciences, Vellore Institute of Technology, Chennai-600048, Tamil Nadu, India.
[3]Centre for Nanodiagnostics of Materials, Faculty of Materials Science and Technology, Slovak University of Technology, Vazovova 5, Bratislava, 812 43.
[4]Institute of Theoretical and Computational Physics, Graz University of Technology, Graz, Austria, 8010
[5]Polymer Institute, Slovak Academy of Sciences, Dúbravská cesta 9, 845 41, Bratislava, Slovakia.
[6]Theory Division, Saha Institute of Nuclear Physics, HBNI, Kolkata, India 700064.

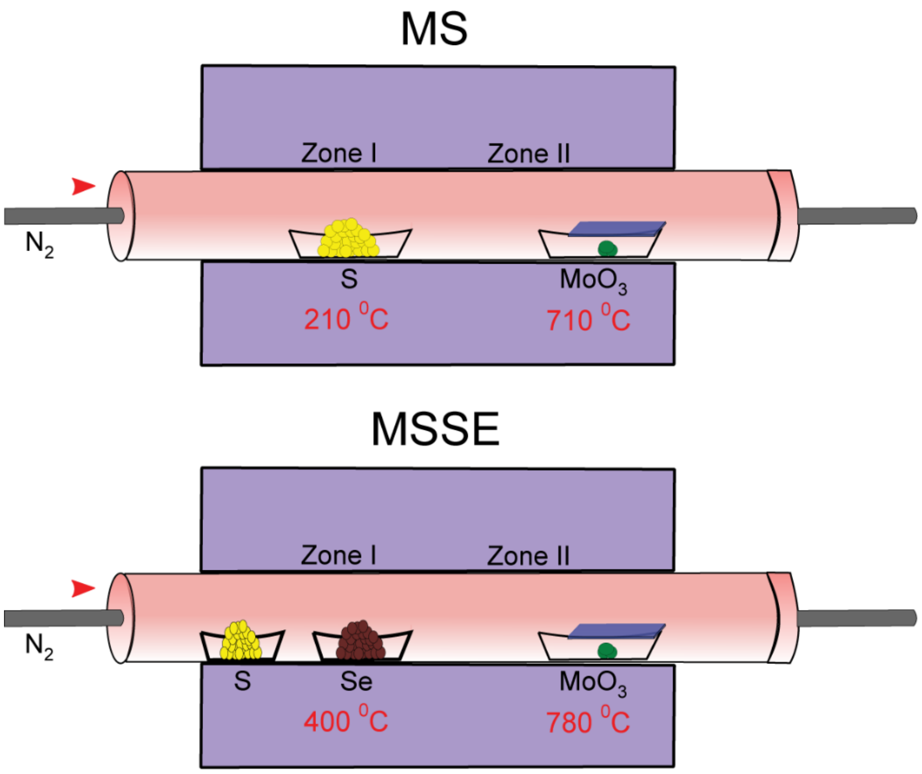

Figure S1: Schematic showing a typical CVD growth of MoS_2 and $MoS_{2(1-x)}Se_{2x}$ 2D atomic layers.

[$]Equally contributed author
[*]Corresponding Author's email: ravikumar.biroju@vit.ac.in; ravi.biroju@stuba.sk

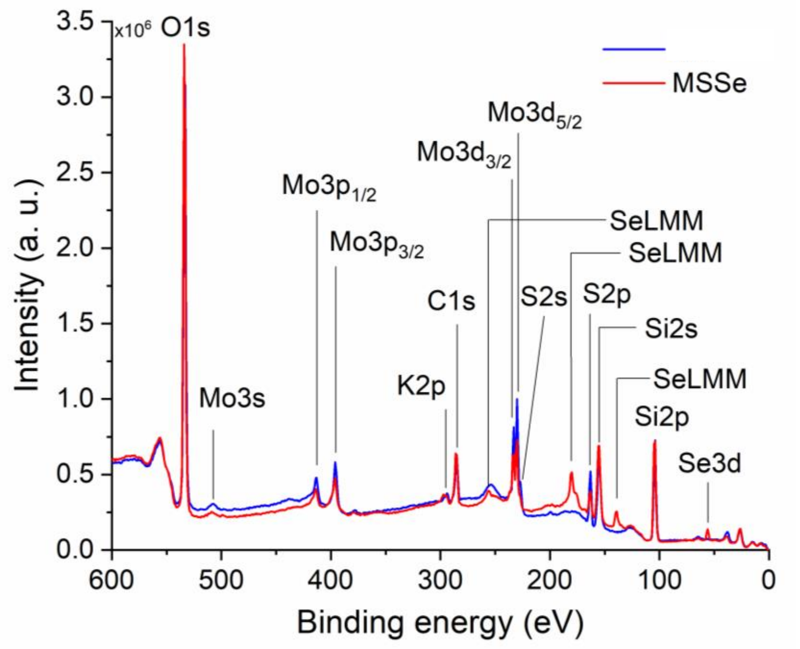

Figure S2: Survey XPS spectra of an as-grown MoS_2 and $MoS_{2(1-x)}Se_{2x}$ alloy.

Table S1: Line shape analysis of XPS spectra shown in Figure 2.

Peak		B.E. (eV) MS / MSSE	*SF*	MS		MSSE	
				peak area	*I*	peak area	*I*
$Se^{(2-)}$	$3d_{5/2}$	--- / 54.7	1.6	---	---	14 913	23 783
$Se^{(2-)}$	$3d_{3/2}$	--- / 55.6		---		8 870	
$S^{(2-)}$	$2p_{3/2}$	162.7 / 162.7	1.881	94 732	131 398	40 064	57 639
$S^{(2-)}$	$2p_{1/2}$	163.9 / 163.9		36 666		17 575	
$Mo^{(4+)}$	$3d_{5/2}$	229.8 / 229.9	11.008	200 201	335 338	132 302	211 341
$Mo^{(4+)}$	$3d_{3/2}$	233.0 / 233.0		135 137		79 039	

QUANTIFICATION for *Xi* (molar fraction of *i*-th element)

$$X_i(\%) = \frac{I_i/SF_i}{\sum_i (I_i/SF_i)} . 100$$

MS

X_{Mo} = 30.4%

X_S = 69.6% → $MoS_{2.29}$

MSSE

X_{Mo} = 29.7%

X_S = 47.4% X_{Se} = 22.9% → $MoS_{1.59}Se_{0.77}$

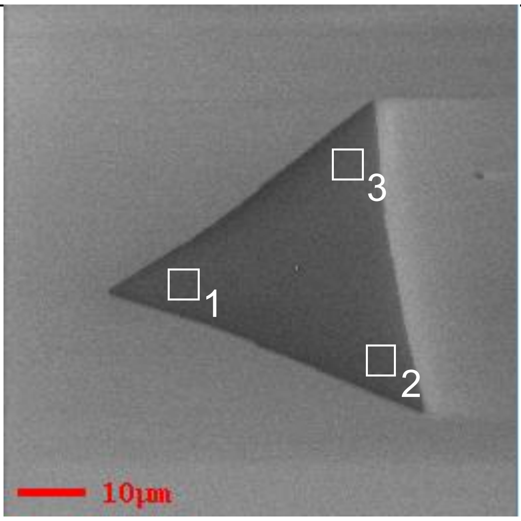
3
1
2
10μm

MS

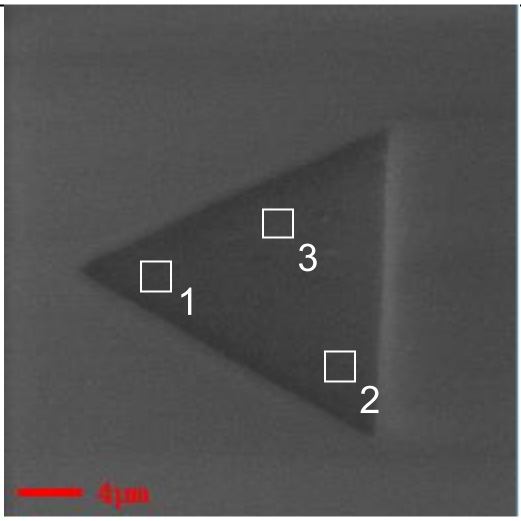
3
1
2

MSSE

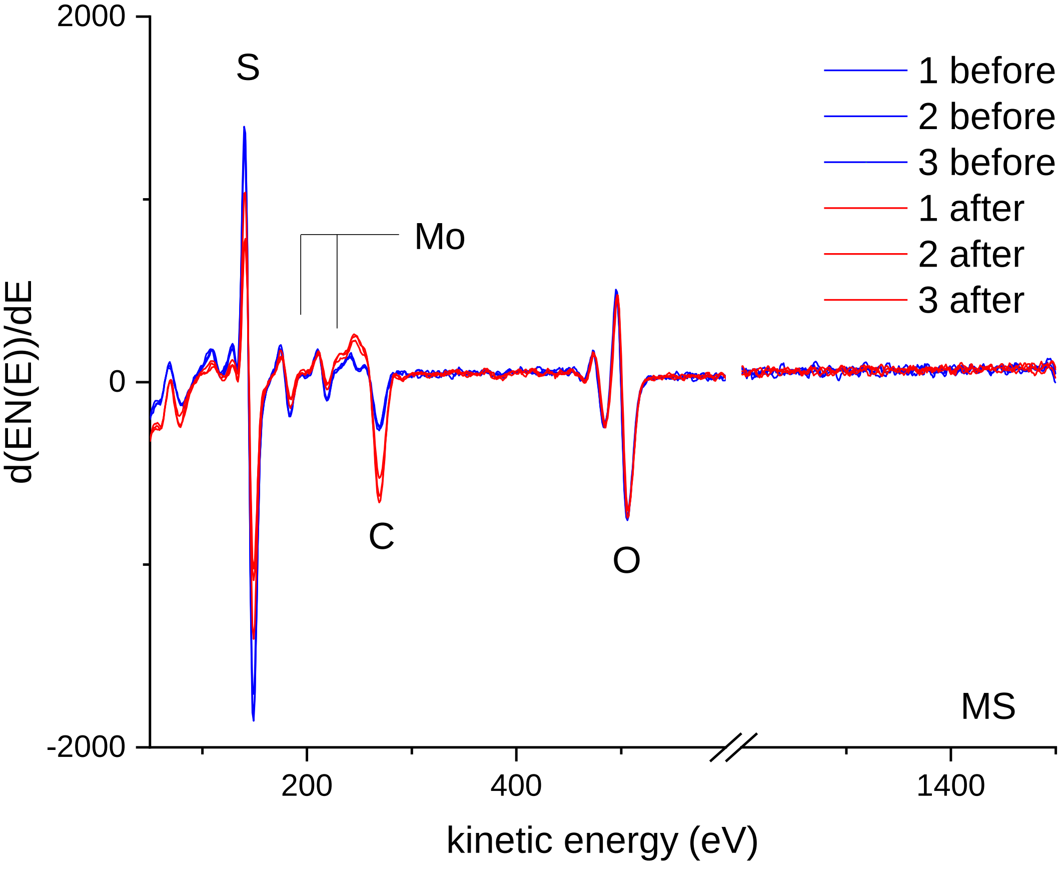
2000
0
-2000
d(EN(E))/dE
S
Mo
C
O
1 before
2 before
3 before
1 after
2 after
3 after
MS
200
400
1400
kinetic energy (eV)

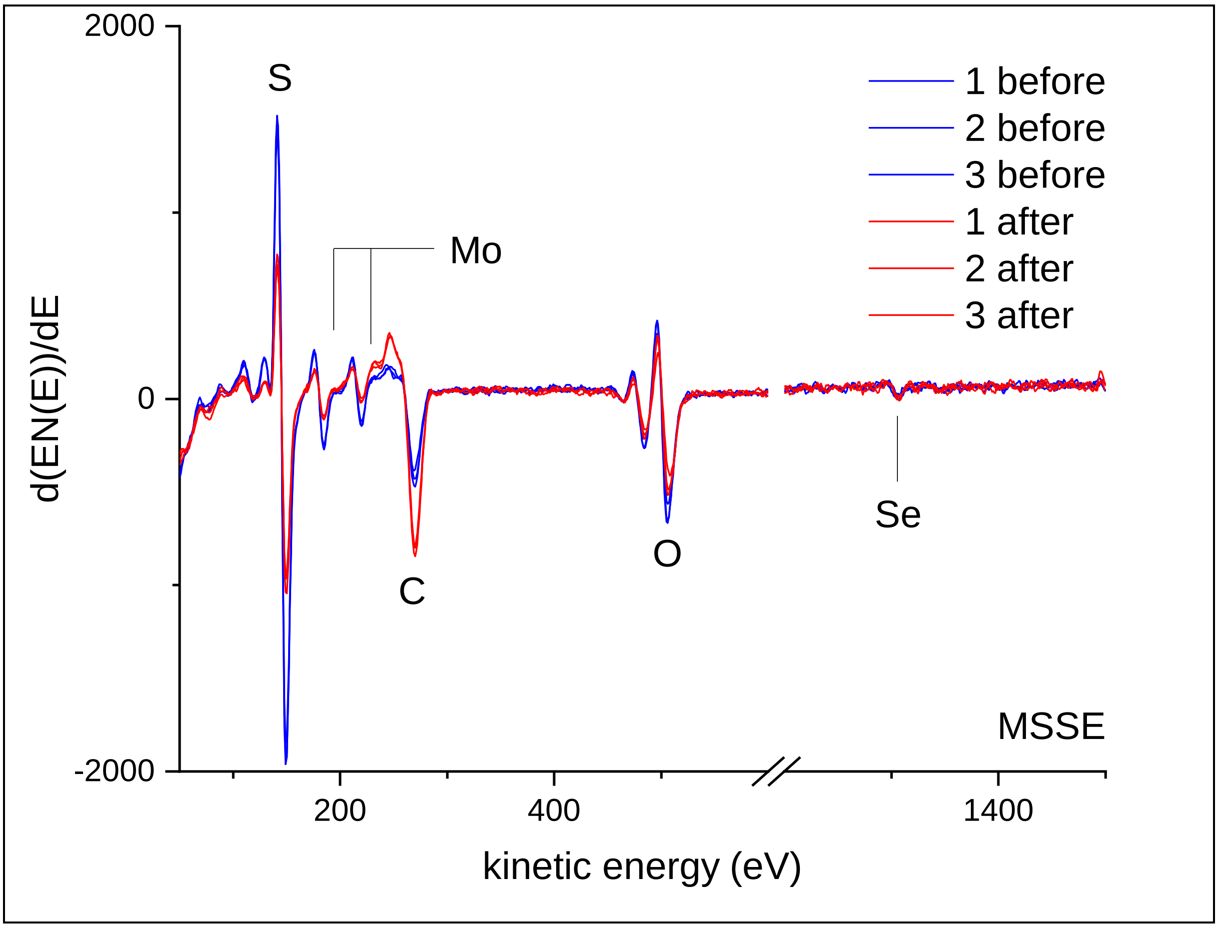

Figure S3: SEM images of MS and MSSE grains with three locations for surface analysis before and after ethanol treatment and corresponding Auger spectra.

Table S2. Intensities of S-LVV and Mo-MNN Auger transitions in the ethanol-treated MS and MSSE samples relative to the corresponding intensities in the untreated samples. Data evaluation is based on the Auger spectra shown in Figure S6.

**Sample**	**MS**			**MSSE**		
**Location**	**1**	**2**	**3**	**1**	**2**	**3**
**S-LVV** (150 eV)	82%	55%	59%	51%	49%	53%
**Mo-MNN** (184 eV)	88%	61%	61%	47%	51%	52%

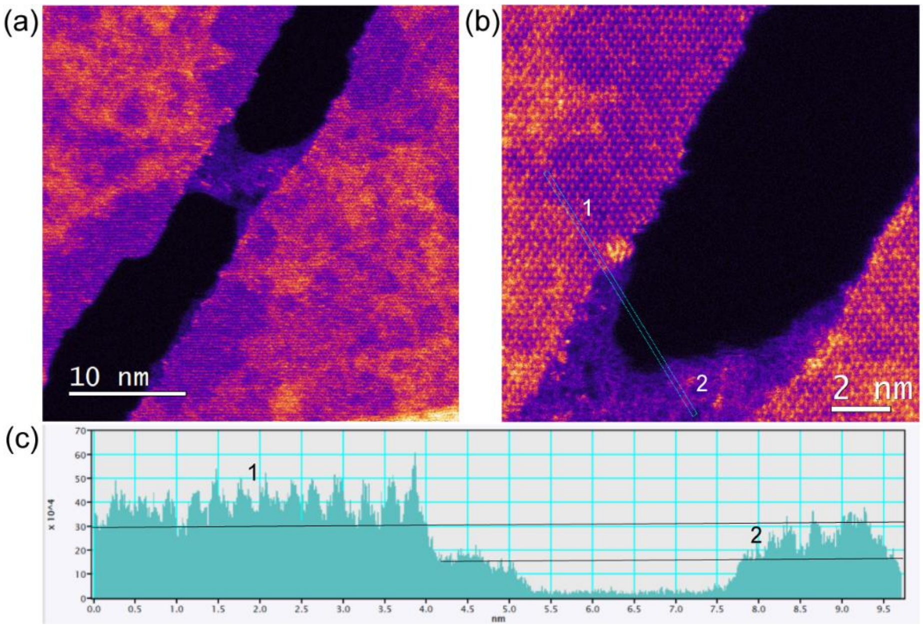

Figure S4: (a-b) HAADF-STEM image of an MSSE before ethanol cleaning, clearly showing the presence of Se atomic sites over the basal plane of MoS_2 and the marked region shows polymer contamination residue, and the higher HAADF intensity species attached with the residual polymer may be the excess precursor elements present on the as-grown MSSE sample. (c) Corresponding atomic intensity line profile of the marked region in Figure (b).

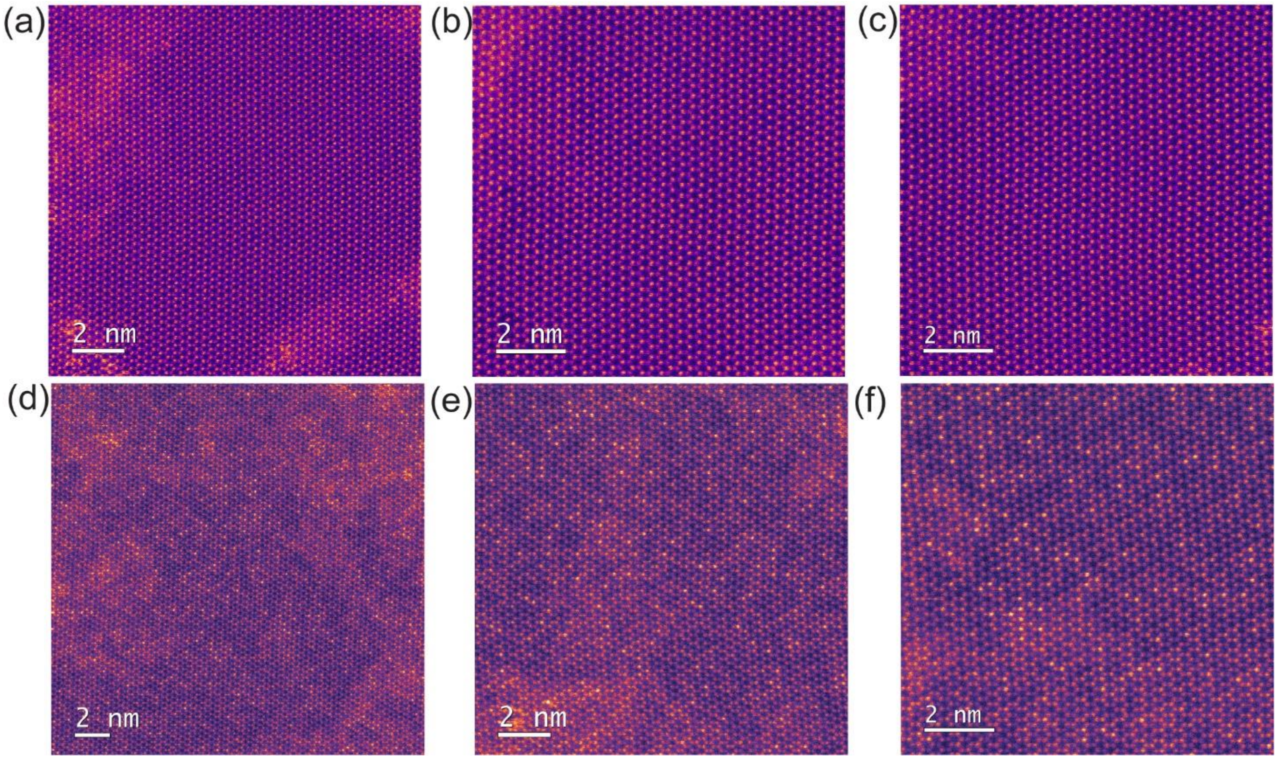

Figure S5: HAADF-STEM images of ethanol-cleaned, as-transferred (a-c) MoS_2 and (d-f) $MoS_{2(1-x)}Se_{2x}$ 2D alloys acquired at different magnifications, such as 10, 15, and 20 Mx, respectively.

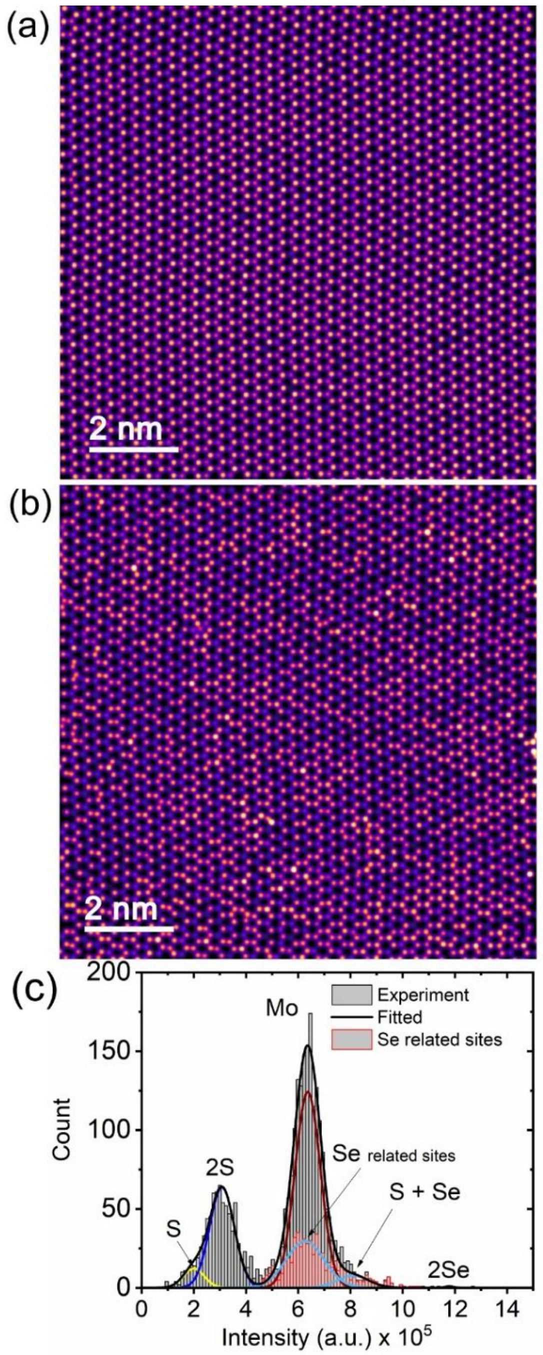

Figure S6: HAADF-STEM image analysis using Stat-STEM software: HAADF-STEM image of (a) MoS_2 and (b) EtOH-MSSE after Gaussian Fitting. Note that the HAADF signal collected from the atomic columns of Mo, S, S_2, S+Se, and Se_2 was found in accordance with the Rutherford scattering cross section proportional to ~ $Z^{1.8}$. Gaussian line shape analysis of EthOH-MSSE shows the fitting of atomic compositions of Mo, S, S_2, Se_2 and S+Se, with a significant shift of the S+SE peak toward the Mo intensity. Note that the chalcogen>2S sites were extracted manually based on atomic intensity higher than that of the two sulfur atoms.

Table S3: Quantitative analysis of atomic sites which is extracted from the Gaussian line shape fitting analysis of HAADF-STEM in Figure 3.

Atom sites		Counts	Density counts /nm^2	Density in %	L_d (nm)
1	Mo	1402	12.79	51.66	0.280
4	S	66	0.96	3.88	1.020
3	S2	764	6.00	24.24	0.408
6	S + Se	567	4.97	20.08	0.449
7	Se2	8	0.02	0.10	6.375
5	Vac	1	0.01	0.04	10.652
Total atoms of S:		2161			
Total atoms of Mo:		1402	Inspected area (nm^2) = 113.47		
Total atoms of Se:		583			
Ratio S + Se : Mo		1.96			
Se concentration %:		21.25			

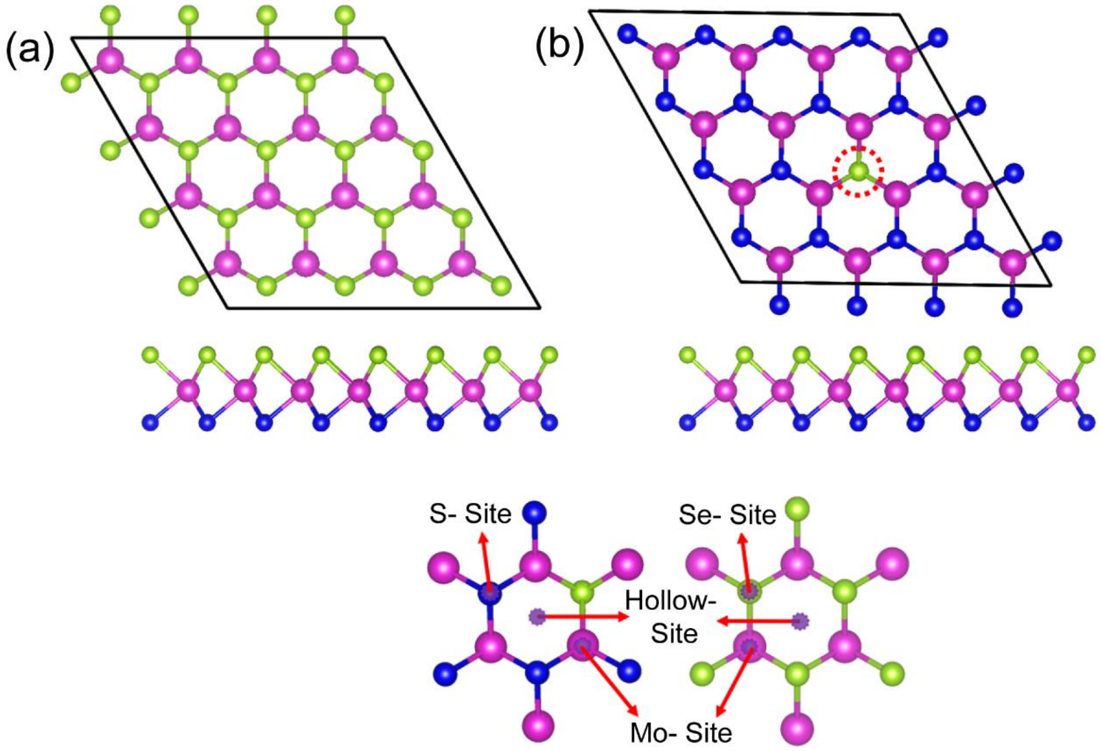

Figure S7: Top and side views of (a) pristine $MoS_{2(1-x)}Se_{2x}$ and (b) sulfur-vacancy $MoS_{2(1-x)}Se_{2x}$ (SV_MSSE) monolayers. (c) Schematic representation of possible adsorption sites on both S- and Se-terminated surfaces. (Mo: magenta, S: blue, Se: green)

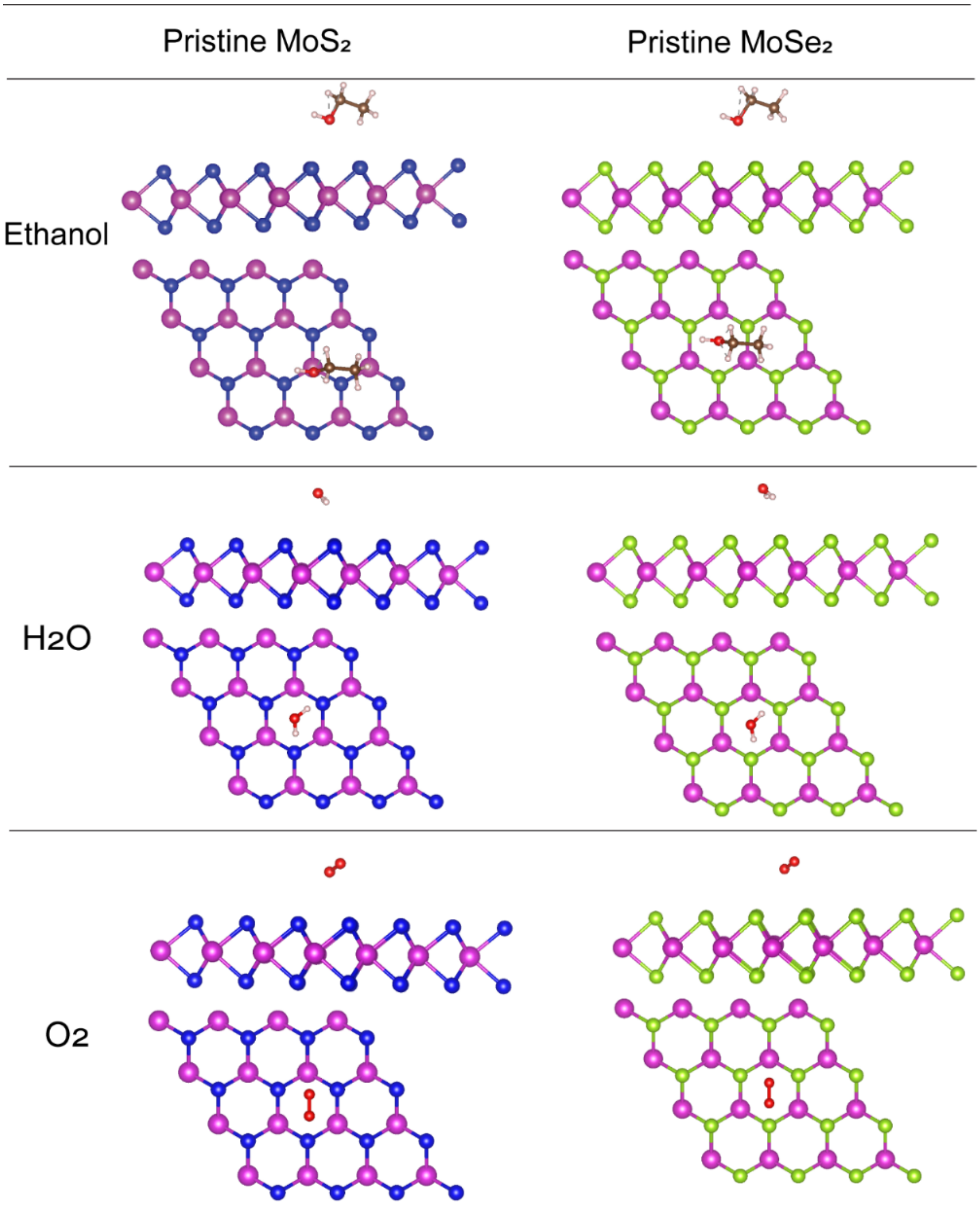

Figure S8: Optimized structures of various molecules (C_2H_5OH) adsorbed on the pristine MoS_2 and $MoSe_2$ monolayers.

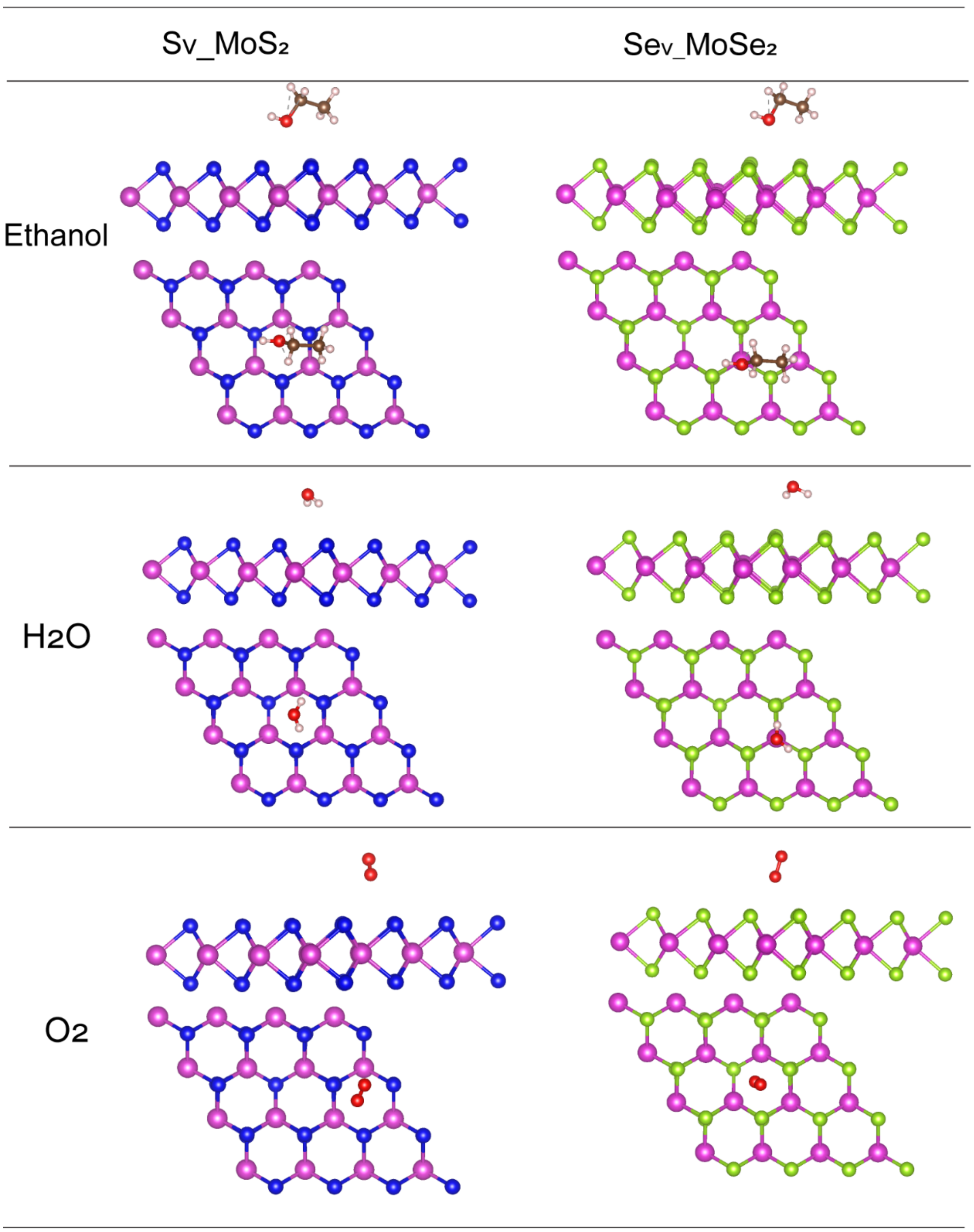

Figure S9: Optimized structures of various molecules (C_2H_5OH) adsorbed on the chalcogen vacancy defect (S_V and Se_V) MoS_2 and $MoSe_2$ monolayers.

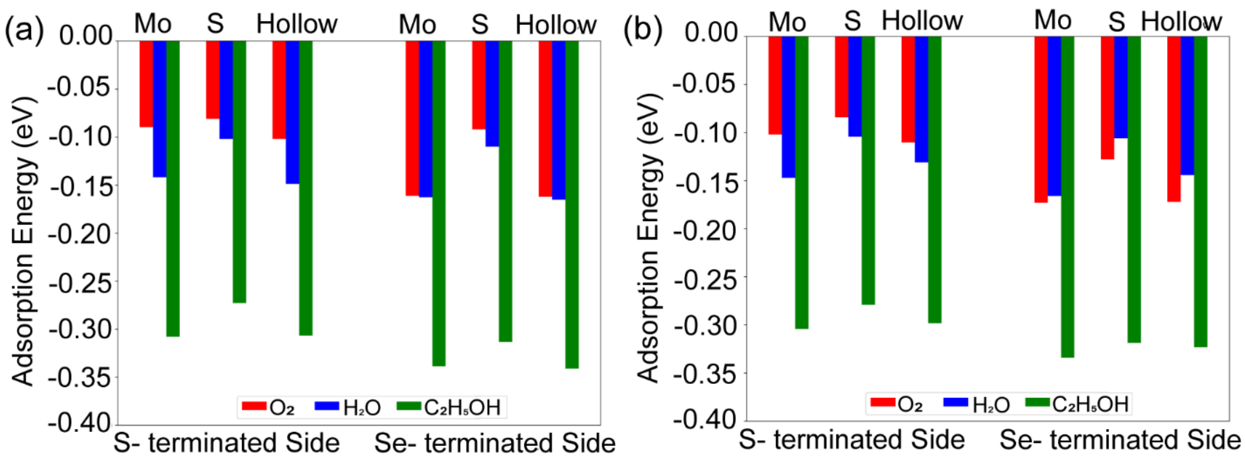

Figure S10: Comparison of adsorption energies of various molecules on (a) pristine and (b) chalcogenide vacancy defect (S_v and Se_V) MoS_2 and $MoSe_2$ monolayers at Mo, chalcogenide (S & Se) and hollow sites.

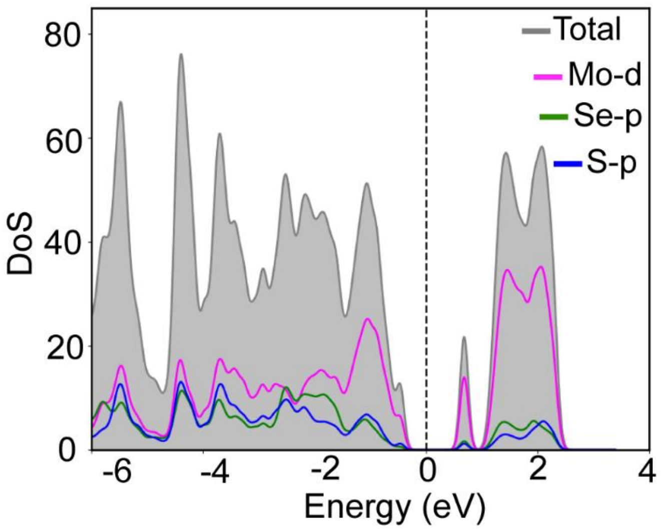

Figure S11: Total and projected density of states of the adsorbate-free MSSE (SV_MSSE) monolayer. The Fermi level is set at 0 eV.
